# Impact of depth of response on survival in patients treated with cobimetinib ± vemurafenib: pooled analysis of BRIM-2, BRIM-3, BRIM-7 and coBRIM

**DOI:** 10.1038/s41416-019-0546-y

**Published:** 2019-08-16

**Authors:** Karl D. Lewis, James Larkin, Antoni Ribas, Keith T. Flaherty, Grant A. McArthur, Paolo A. Ascierto, Brigitte Dréno, Yibing Yan, Matthew Wongchenko, Edward McKenna, Qian Zhu, Yong Mun, Axel Hauschild

**Affiliations:** 1 Department of Medicine, University of Colorado Comprehensive Cancer Center, Aurora, CO 80045 USA; 2 Skin Unit, The Royal Marsden NHS Foundation Trust, London, SW3 6JJ UK; 3 Departments of Medicine and Hematology and Oncology, Jonsson Comprehensive Cancer Center at the University of California, Los Angeles, Los Angeles, CA 90095 USA; 4 Department of Medicine, Massachusetts General Hospital, Boston, MA 02114 USA; 5 Peter MacCallum Cancer Centre, Melbourne, VIC 3000 Australia; 6 Department of Oncology, University of Melbourne, Parkville, VIC 3000 Australia; 7 Cancer Immunotherapy and Innovative Therapy Unit, Istituto Nazionale Tumori Fondazione G. Pascale, Naples, 80131 Italy; 8 Department of Oncology, Nantes University, Nantes, 44093 France; 9 Product Development Oncology, Genentech, Inc., South San Francisco, CA USA; 10 Medical Affairs, Genentech, Inc., South San Francisco, CA 94080 USA; 11 Department of Dermatology, University Hospital Schleswig-Holstein, Kiel, D-24105 Germany

**Keywords:** Melanoma, Prognostic markers, Prognostic markers, Melanoma

## Abstract

**Background:**

This pooled analysis investigated the prognostic value of depth of response in two cohorts of patients with *BRAF*^V600^-mutated metastatic melanoma treated with vemurafenib or cobimetinib plus vemurafenib.

**Methods:**

The data were pooled from BRIM-2, BRIM-3, BRIM-7 and coBRIM. Association of depth of response with survival was estimated by Cox proportional hazards regression, adjusted for clinically relevant covariates. Depth of response was analysed in previously identified prognostic subgroups based on disease characteristics and gene signatures.

**Results:**

Greater tumour reduction and longer time to maximal response were significantly associated with longer progression-free survival (PFS) and overall survival (OS) when evaluated as continuous variables. Patients with the deepest responses had long-lasting survival outcomes (median PFS: 14 months; OS: 32 months with vemurafenib; not estimable with cobimetinib plus vemurafenib). Cobimetinib plus vemurafenib improved depth of response versus vemurafenib monotherapy regardless of other prognostic factors, including gene signatures.

**Conclusions:**

Greater depth of response was associated with improved survival, supporting its utility as a measure of treatment efficacy in melanoma and further evaluation of its incorporation into existing prognostic models. Cobimetinib plus vemurafenib improved outcomes across quartiles of response regardless of prognostic factors or gene signatures and provided durable survival benefits in patients with deep responses.

## Background

The introduction of small-molecule BRAF inhibitors has improved progression-free (PFS) and overall survival (OS) for patients with *BRAF*^V600^-mutated metastatic melanoma.^1–3^ However, resistance to BRAF inhibitors due to reactivation of the mitogen-activated protein kinase (MAPK) pathway through MEK signalling limits the median duration of PFS to ~6 months.^4,5^ Combined BRAF and MEK inhibition provides broader and more comprehensive inhibition of the MAPK pathway than BRAF inhibition alone, and combination therapy with cobimetinib (a MEK inhibitor) and vemurafenib (a BRAF inhibitor) has demonstrated significant clinical benefit compared with vemurafenib monotherapy.^5,6^ Combined BRAF- and MEK-targeted therapy is now a standard of care in patients with *BRAF*^V600^-mutated melanoma.

Responses to BRAF and/or MEK inhibition vary among patients, and it would be useful to identify those who respond well to targeted therapy. Post hoc analyses of pooled data from multiple trials suggest that greater depth of response is associated with better disease control and increased survival in patients with haematologic^7^ and solid cancers.^8,9^ Depth of response is a more personalised and granular measure of tumour response than Response Evaluation Criteria in Solid Tumours (RECIST) categories of response, which could be helpful in assessing the variation in patient outcome following initiation of targeted therapy. The question remains whether the individual patients who have the greatest reduction in tumour burden (depth of response) are the patients who have the greatest OS or PFS.^9^

In this exploratory analysis, we aimed to evaluate the association of depth of tumour response with survival outcomes in two cohorts of patients treated with vemurafenib monotherapy or cobimetinib plus vemurafenib. Prognostic subgroups defined by particular disease characteristics and gene expression signatures that affect survival outcomes have been identified in patients with *BRAF*^V600^-mutated metastatic melanoma treated with BRAF and MEK inhibitors.^10–12^ We additionally evaluated the association between depth of response and survival outcomes within these prognostic subgroups.

## Methods

### Analysis population

The data were pooled from the BRIM-2, BRIM-3, BRIM-7 and coBRIM studies. Detailed methods have been previously reported.^1,2,5,6^ Briefly, BRIM-2 (ClinicalTrials.gov ID, NCT00949702) was an open-label, multicentre phase 2 trial of oral vemurafenib.^2^ BRIM-3 (ClinicalTrials.gov ID: NCT01006980) was an open-label, multicentre, randomised phase 3 trial of oral vemurafenib versus intravenous dacarbazine.^1,13^ BRIM-7 (ClinicalTrials.gov ID: NCT01271803) was an open-label, multicentre phase 1b dose-escalation study of oral cobimetinib plus oral vemurafenib at various doses.^6^ coBRIM (ClinicalTrials.gov ID, NCT01689519) was a randomised, double-blind phase 3 study of oral cobimetinib plus oral vemurafenib compared with placebo and vemurafenib.^5,14^ Key eligibility criteria were similar across trials, including age ≥ 18 years, unresectable stage IIIC or IV *BRAF*^V600^-mutated melanoma, Eastern Cooperative Oncology Group performance status (ECOG PS) of 0–1, and adequate organ function. BRIM-3 and coBRIM enrolled previously untreated patients only, whereas BRIM-2 enrolled patients who had received prior systemic treatment for advanced disease. Only BRAF inhibitor–naive patients from BRIM-7 were included in this analysis. All four trials allowed enrolment of patients with previously treated, stable brain metastases. All analysed patients received vemurafenib 960 mg orally twice daily with or without cobimetinib 60 mg orally daily for 21 days followed by a 7-day break.

Each trial was conducted in accordance with the Declaration of Helsinki and the principles of Good Clinical and Laboratory Practice and with the approval of appropriate ethics committees. Ethical approval of the clinical studies included in this analysis were provided by the local Institutional Review Boards (IRBs), and a list of IRBs approving each study is documented in the online Supplementary IRB List. All participants provided written informed consent.

### Depth of response

Depth of response was defined by maximal tumour reduction or time to maximal tumour reduction. Tumour reduction (Max%SLD) is defined as the greatest reduction or minimum increase in the sum of longest diameters of the target lesion (SLD) from baseline to the first progression of disease (PD) or date of last follow-up. Max%SLD is calculated as ([smallest SLD measured before the first PD–baseline SLD]/baseline SLD) × 100. Time to maximal change in tumour size (TimeMax%SLD) is defined as the time from the start of study treatment or randomisation to the date of reaching the maximum change in SLD before or at the first PD. To demonstrate the relationship with tumour reduction across the continuum of response, from complete response (CR) to PD, each cohort was rank-ordered for Max%SLD and divided into quartiles for Kaplan–Meier analysis of survival outcomes.

### Gene expression signatures

Gene expression signatures associated with PFS were previously identified using archival formalin-fixed, paraffin-embedded tumour samples from patients in the BRIM-2, BRIM-3, BRIM-7 and coBRIM trials (see Supplementary Data for the full list of genes in each signature).^10^ Patients’ gene expression data, measured using the nCOUNTER platform (NanoString Technologies, Seattle, WA), were analysed in relation to depth of response variables by Cox proportional-hazards modelling.

### Statistical analysis

The primary end points for this analysis were PFS and OS, estimated using the Kaplan–Meier method. The association of depth of response with PFS and OS outcomes was tested by multivariate Cox proportional-hazards modelling adjusted for clinically relevant covariates (age, sex, race, geographic region, ECOG PS, lactate dehydrogenase levels, disease stage, presence/absence of liver metastasis, SLD of target lesions, Max%SLD and TimeMax%SLD). Max%SLD and TimeMax%SLD were analysed as continuous covariates in the proportional-hazards model, with units of difference of 1% and 1 month, respectively. Time-dependent multivariate Cox proportional-hazards modelling was conducted as a sensitivity analysis.

All analyses were conducted using data cut-off dates of 1 February 2012 for BRIM-2; 8 July 2015 for BRIM-3; 2 December 2015 for BRIM-7 and 28 August 2015 for coBRIM, except for the Kaplan–Meier analysis of survival outcomes and landmark survival rates by tumour reduction quartiles, for which the data cut-off dates of 10 July 2017 (BRIM-7) and 13 October 2017 (coBRIM) were used. All eligible patients, as defined by each study protocol, were included in the analysis regardless of treatment assignment.

## Results

### Patient demographics

Baseline characteristics were similar between the vemurafenib monotherapy pooled cohort and the cobimetinib plus vemurafenib pooled cohort (Table S1). In both cohorts, ~45% of patients had elevated lactate dehydrogenase (LDH) levels, a marker of poor prognostic outcomes.^15^ Approximately one-third had liver metastases at baseline. The median baseline lesion size (sum of longest diameters) was 68 mm for the vemurafenib monotherapy pooled cohort, and 63 mm for the cobimetinib plus vemurafenib pooled cohort.

### Depth of response correlation to survival outcomes

Maximal tumour reduction and time to best response were significantly associated with PFS and OS when evaluated as continuous variables (Table 1). For every 1% increase in maximal tumour reduction within each cohort, there was an incremental 1–2% decrease in the relative risk of death or progression for vemurafenib monotherapy and cobimetinib plus vemurafenib, respectively, which was statistically significant (Table 1). For every month increase in time to maximal response, the relative risk of progression was decreased by 14% or 16%, and the relative risk of death was decreased by 12% or 15% for vemurafenib monotherapy and cobimetinib plus vemurafenib, respectively (*p* < 0.001 for all; Table 1). Sensitivity analyses with time-dependent multivariate Cox proportional-hazards regression modelling confirmed the independent association of tumour reduction with survival outcomes. For time to best response, time-dependent Cox modelling confirmed an association with OS but not with PFS (Table S2). Similar findings were observed with the analysis conducted in the subgroup of patients with gene signature profiles and adjusted for signature profile (Table S3).

**Table 1:** Cox proportional-hazards analysis of survival outcomes with depth-of-response variables in pooled patient cohorts

Survival outcome	Pooled V cohort, *n* = 717	Pooled C + V cohort, *n* = 310
*PFS*		
Event, *n*	609	203
K–M median, months (95% CI)	6.9 (6.2–7.0)	12.7 (10.6–14.7)
HR (95% CI)^a^	1.0 (reference)	0.80 (0.67–0.95)
* p-*value^a^		0.0103
Max%SLD before/at first PD		
HR per 1% increase in Max%SLD (95% CI)^b^	1.01 (1.01–1.01)	1.02 (1.01–1.02)
* p-*value^b^	<0.0001	<0.0001
TimeMax%SLD before/at first PD		
HR per 1 month increase in TimeMax%SLD (95% CI)^b^	0.86 (0.84–0.88)	0.84 (0.81–0.87)
* p-*value^b^	<0.0001	<0.0001
*OS*		
Event, *n*	495	148
K–M median, months (95% CI)	15.0 (13.8–16.8)	28.0 (21.8–31.2)
HR (95% CI)^a^	1.0 (reference)	0.84 (0.68–1.03)
* p-*value^a^		0.0909
Max%SLD before/at first PD		
HR per 1% increase in Max%SLD (95% CI)^b^	1.01 (1.00–1.01)	1.01 (1.00–1.01)
* p-*value^b^	0.0003	0.0071
TimeMax%SLD before/on first PD		
HR per 1 month increase in TimeMax%SLD (95% CI)^b^	0.88 (0.85–0.91)	0.85 (0.81, 0.89)
* p-*value^b^	<0.0001	<0.0001

*C* + *V* cobimetinib plus vemurafenib, *CI* confidence interval, *HR* hazard ratio, *K–M* Kaplan–Meier, *Max%SLD* maximum percentage change in the sum of longest diameters, *OS* overall survival, *PD* disease progression, *PFS* progression-free survival, *SLD* sum of longest diameters, *TimeMax%SLD* time to maximum percentage change in the sum of longest diameters, *V* vemurafenib monotherapy

^a^The HR and *p-*values from the Cox proportional hazards regression model are for the comparison of two-pooled cohorts (pooled cobimetinib plus vemurafenib versus pooled vemurafenib)

^b^The HR and *p-*values from the Cox proportional hazards regression model are for Max%SLD of target lesions before or on the first PD, or TimeMax%SLD of target lesion before or at the first PD within each cohort, analysed as continuous variables within each cohort

**Fig. 1:**
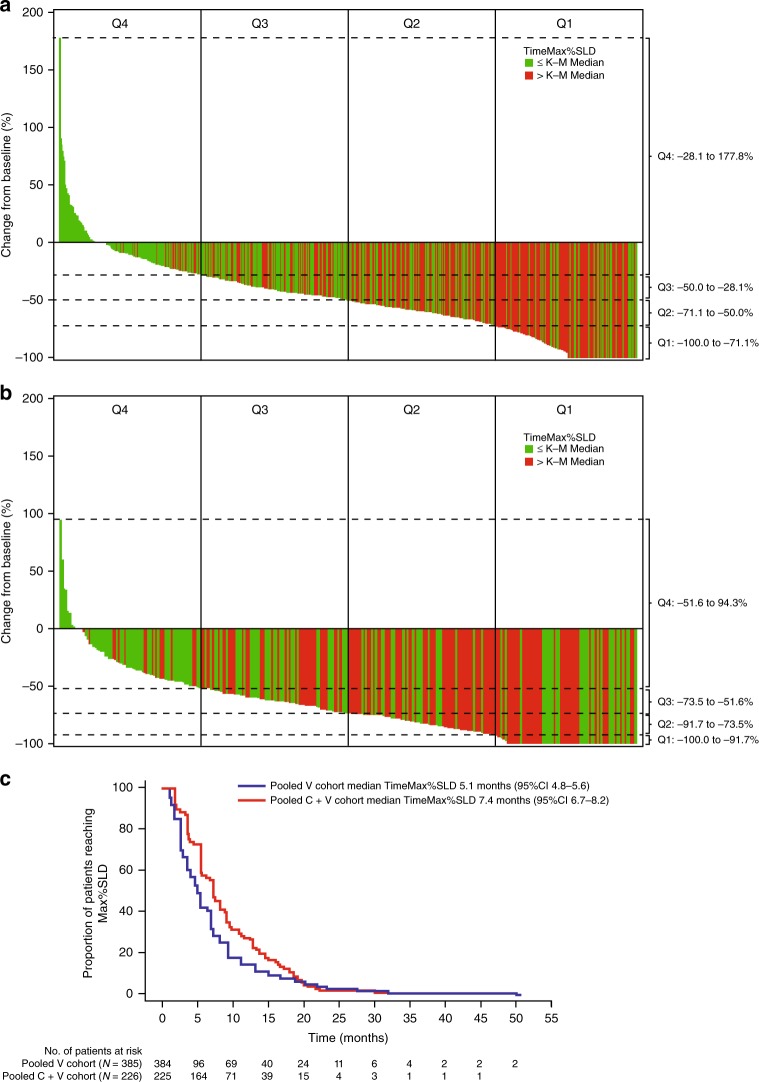
Waterfall plots of tumour reduction and time to best response in (**a**) vemurafenib monotherapy cohort and (**b**) cobimetinib plus vemurafenib pooled cohort, and (**c**) Kaplan–Meier median time to best response in patients who had a response to treatment. Bars are colour-coded to indicate whether time to best response is longer (red) or shorter (green) than the median. Dashed lines indicate the tumour reduction ranges for the quartiles used in the analyses. C + V cobimetinib plus vemurafenib, K–M Kaplan–Meier, Max%SLD maximum percentage change in the sum of longest diameters, PD progression of disease, Q quartile, TimeMax%SLD time to maximum percentage change in the sum of longest diameters, V vemurafenib

Maximal tumour reduction and time to best response were greater in the cobimetinib plus vemurafenib pooled cohort (Fig. 1a–c). In the vemurafenib monotherapy pooled cohort (Fig. 1a), 91.9% patients experienced tumour reduction, with a median reduction of −53.3%. In the cobimetinib plus vemurafenib pooled cohort (Fig. 1b), a slightly greater proportion of patients (95.9%) experienced tumour reduction, and the median reduction was greater (−74.4%). Across treatment cohorts, patients with deep responses (Q1; Fig. 1a, b) generally had a time to maximal response greater than the median: 5.1 and 7.4 months for the vemurafenib and cobimetinib plus vemurafenib cohorts, respectively (Fig. 1c). This observation was particularly true for the vemurafenib cohort. Thus, patients with a greater degree of tumour reduction (Q1/Q2) appeared more likely to have a time to best response that was longer than the median (Fig. 1a, b), while patients with little or no tumour reduction typically had a time to best response that was shorter than the median.

**Fig. 2:**
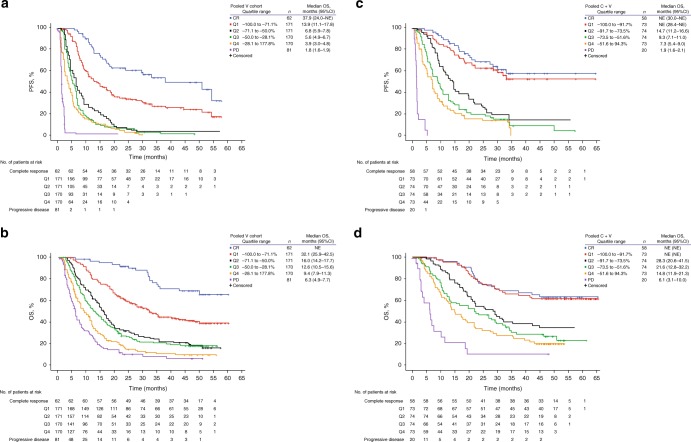
Kaplan–Meier analysis of (**a**) PFS and (**b**) OS by tumour reduction quartiles, and in patients with complete response or disease progression, in the vemurafenib monotherapy cohort, and of (**c**) PFS and (**d**) OS in the cobimetinib plus vemurafenib cohort. CI confidence interval, CR complete response, C + V cobimetinib plus vemurafenib, NE not estimable, OS overall survival, PD progression of disease, PFS progression-free survival, Q quartile, V vemurafenib

When grouped into quartiles based on tumour reduction (Fig. 1a, b), patients with the deepest responses (Q1 in each cohort) had better survival outcomes than those in Q2–Q4. Median PFS in Q1 of the vemurafenib monotherapy pooled cohort was 13.9 months (95% confidence interval [CI]: 11.1–17.8 months); median OS was 32.1 months (95% CI: 25.9–42.5 months) (Fig. 2a, b). Median PFS and OS for patients in Q1 of the cobimetinib plus vemurafenib pooled cohort were not reached at the time of analysis (Fig. 2c, d). The majority of patients within Q1 of the cobimetinib plus vemurafenib pooled cohort experienced a CR (67%, *n* = 49/73), whereas in Q1 of the vemurafenib monotherapy cohort, fewer patients experienced a CR (35%, *n* = 60/171), although the relationship between survival and depth of response was maintained across cohorts. Further analysis of relationship to landmark PFS and OS rates, which standardised quartiles across treatment cohorts to control for the differences in distribution of maximal tumour reduction and quartile ranges between the treatment cohorts, found better survival outcomes in the cobimetinib plus vemurafenib pooled cohort than in the vemurafenib monotherapy pooled cohort across all Max%SLD quartiles, regardless of the quartile definitions used (Fig. S1a–d).

### Depth of response in relation to prognostic factors

Subgroups of patients with *BRAF*-mutated melanoma treated with BRAF and MEK inhibitors are associated with distinct survival outcomes, based on clinical characteristics (LDH levels, ECOG PS, presence of liver metastasis and tumour size [SLD]).^11,12^ When patients in the pooled treatment cohorts were sorted into these prognostic subgroups, we found greater median tumour reduction in subgroups associated with good prognosis and lesser median tumour reduction in subgroups associated with poor prognosis (Table 2). In the cohort of patients treated with cobimetinib plus vemurafenib, treatment effect as assessed by maximal tumour reduction was less influenced by poor prognostic factors than in the vemurafenib monotherapy cohort.

**Table 2:** Tumour reduction in PFS prognostic subgroups in the vemurafenib monotherapy pooled cohort and the cobimetinib plus vemurafenib pooled cohort

Prognostic subgroup	Max%SLD in pooled V cohort			Max%SLD in pooled C + V cohort		
	*n*	Median	IQR	*n*	Median	IQR
All pooled patients with tumour reduction^a^	627	–53.3	–73.6 to –33.8	282	–74.4	–94.1 to –55.2
LDH level normal and liver metastases absent	266	–56.8	–88.6 to –38.2	117	–75.0	–100 to –56.3
LDH level normal and liver metastases present	87	–61.1	–77.2 to –43.4	35	–75.0	–90.4 to –51.7
LDH level elevated and ECOG PS = 0	121	–47.5	–69.1 to –30.3	85	–77.0	–91.0 to –61.8
LDH elevated and ECOG PS > 0	148	–45.7	–59.9 to –27.7	39	–60.0	–75.4 to –46.2

*C* + *V* cobimetinib plus vemurafenib, *ECOG PS* Eastern Cooperative Oncology Group performance status, *IQR* interquartile range, *LDH* lactate dehydrogenase, *Max%SLD* maximum percentage change in the sum of longest diameters, *V* vemurafenib monotherapy

^a^Includes only patients with negative Max%SLD of target lesion

In patients with *BRAF*-mutated metastatic melanoma treated with BRAF and MEK inhibitors, certain gene signatures have been identified as prognostic (Table S4)^10,16^—the ‘immune’ gene signature being favourable and the ‘cell cycle’ being unfavourable. Among patients with gene signature data, those in the cobimetinib plus vemurafenib pooled cohort had a greater maximal tumour reduction, objective response rate, CR rate and a longer duration of response than those in the vemurafenib monotherapy pooled cohort, regardless of gene signature type (Table 3). This difference was more marked in patients expressing the cell cycle gene signature in their tumours at baseline than in patients expressing the immune gene signature.

**Table 3:** Objective responses by pooled cohort for all patients with gene expression signature data

Variable	Patients with immune gene signature		Patients with cell cycle gene signature	
	Pooled V cohort (*n* = 148)	Pooled C + V cohort (*n* = 72)	Pooled V cohort (*n* = 172)	Pooled C + V cohort (*n* = 71)
Responders, *n*	97	54	77	52
*Response rate, % (95% CI)*				
CR	16.2 (10.7, 23.2)	19.4 (11.1, 30.5)	5.2 (2.4, 9.7)	14.1 (7.0, 24.4)
PR	49.3 (41.0, 57.7)	55.6 (43.4, 67.3)	39.5 (32.2, 47.3)	59.2 (46.8, 70.7)
SD	23.0 (16.5, 30.6)	13.9 (6.9, 24.1)	38.4 (31.1, 46.1)	18.3 (10.1, 29.3)
PD	8.1 (4.3, 13.7)	8.3 (3.1, 17.3)	12.2 (7.7, 18.1)	7.0 (2.3, 15.7)
Median Max%SLD (IQR)	−55.6 (−81.6, −33.3)	−66.1 (−89.5, −45.5)	−44.8 (−63.7, −20.0)	−72.4 (−91.0, −51.9)
Median duration of response, months (95% CI)	7.6 (5.8, 13.2)	13.5 (9.5, NE)	7.1 (5.7, 8.3)	11.7 (9.3, 19.8)
HR (95% CI)^a^		0.48 (0.31, 0.76)		0.53 (0.33, 0.84)
*p-*value^a^		0.0014		0.0074

*C* *+* *V* cobimetinib plus vemurafenib, *CI* confidence interval, *CR* complete response, *ECOG PS* Eastern Cooperative Oncology Group performance status, *HR* hazard ratio, *IQR* interquartile range, *LDH* lactate dehydrogenase, *Max%SLD* maximum percentage change in the sum of longest diameters, *NE* not estimable, *PD* progression of disease, *PR* partial response, *SD* stable disease, *V* vemurafenib monotherapy

^a^Cox proportional-hazards regression model with pooled cohort adjusting for age, sex, race, geographic region, ECOG PS, LDH, disease stage, liver metastases, sum of longest diameters of target lesion. The HR and *p-*values are for the comparison of the pooled cobimetinib plus vemurafenib cohort versus the pooled vemurafenib monotherapy cohort

## Discussion

In this exploratory analysis, greater tumour reduction was consistently and independently associated with improved survival outcomes when other known prognostic variables were taken into account. These data suggest that while initial responses occur quickly, deep responses are associated with longer time on treatment and continue to develop over time. Association of time to maximal response with survival outcomes was less consistent, likely due to inherent bias. Cobimetinib plus vemurafenib improved survival outcomes versus vemurafenib monotherapy across maximal tumour reduction quartiles and clinical prognostic subgroups. Patients with the deepest responses experienced a long-term benefit from combined cobimetinib and vemurafenib with 3-year landmark survival outcome rates comparable with long-term survival reported with immunotherapies.^17^ Depth of response has been associated with survival outcomes in other solid tumour settings,^8,9,18^ and, in combination with these data, suggests that depth of response is a meaningful indicator of therapeutic activity and could identify patients with the best chance of long-term survival. A previous analysis that included a subset of patients from the current analyses (i.e., patients enrolled in the BRIM-2 and BRIM-3 studies) found no correlation between early response at 12 weeks and OS in patients with melanoma treated with vemurafenib or dacarbazine.^19^ However, it is important to note that the results of this analysis would have been confounded by the use of a defined time point, creating heterogeneous groups of patients within each response group. In the current analysis, median time to maximal tumour reduction was 5.1 and 7.4 months with vemurafenib and with cobimetinib plus vemurafenib, respectively; thus, while some patients may have already achieved maximal response at 12 weeks, a substantial proportion would be expected to have continued tumour shrinkage beyond this time point. In the current analysis, we evaluated depth of response as a continuous variable (maximal tumour reduction or time to maximal response), thereby minimising heterogeneity and reducing confounding.

Depth of response, particularly maximal tumour reduction, appears to provide additional prognostic/predictive information beyond that obtained from traditional clinical variables (such as elevated LDH levels and measures of tumour burden), or baseline tumour gene expression signature. These can be assessed prior to the initiation of therapy and used to counsel patients about their expected outcomes. Depth of response appears to refine prognostication once response assessments are performed. Gene expression signature has independent prognostic significance for survival outcomes when incorporated into recursive partition models using clinical variables.^16^ The qualitative differences in maximal tumour reduction observed between the immune and cell cycle gene signatures may explain equalisation of maximal tumour reduction across clinical prognostic groups in the cobimetinib plus vemurafenib cohort versus the vemurafenib cohort, given the higher prevalence of the ‘cell cycle’ gene signature in unfavourable clinical prognostic groups.^16^

The strengths of this exploratory analysis include a large patient sample with sufficient survival follow-up for robust statistical analysis across prognostic subgroups, including gene signatures, made possible by pooling patients across a series of studies with similar populations. Limitations include the comparison having been carried out post-randomisation, so that potentially important prognostic values are not balanced between quartiles, the influence of immortal time bias associated with time-dependent outcomes, and the OS end point potentially being confounded by post-progression treatments. Sensitivity analyses of time-dependent Cox proportional-hazards regression modelling were performed to address the potential confounding factor of immortal time bias in this type of analysis,^20^ which confirmed the independent association of tumour reduction with survival outcomes.

In conclusion, depth of response particularly as measured by maximal tumour reduction is associated with survival outcomes, independent of gene signatures or other clinical prognostic factors. Depth of response may provide prognostic/predictive value in addition to traditional clinical prognostic variables, and merits further evaluation in integrated prognostic/predictive models based on this analysis as well as other reported analyses. Incorporation of on-treatment data could further refine prognostic models that may help with individual treatment decisions. In this exploratory analysis, cobimetinib plus vemurafenib improved outcomes across response quartiles regardless of clinical prognostic factors or gene signatures and provided durable survival benefits in patients with the deepest responses. These findings provide further support for combination BRAF and MEK inhibition as a standard of care in this disease setting.

## Supplementary information

Supplemental Tables and Figures

Supplemental IRB list

## Data Availability

The authors declare that all data supporting the findings of this study are available within the article and its Supplementary Information files or are available from the corresponding author upon reasonable request.
