# Supplementary material for: Impact of depth of response on survival in patients treated with cobimetinib ± vemurafenib: pooled analysis of BRIM-2, BRIM-3, BRIM-7 and coBRIM
Source: Br J Cancer. 2019 Aug 16;121(7):522–8. doi: 10.1038/s41416-019-0546-y (PMC6889491; doi:10.1038/s41416-019-0546-y)
Supplement: Supplementary file 1 — Supplemental Tables and Figures [file 41416_2019_546_MOESM1_ESM.docx]

**Table S1.** **Demographics and baseline characteristics of pooled cohorts**

| **Characteristic** | **Pooled V cohort**  ***n* = 717** | **Pooled C + V cohort**  ***n* = 310** |
| --- | --- | --- |
| Age, *n* (%) |  |  |
| <65 y | 530 (73.9) | 232 (74.8) |
| ≥65 y | 187 (26.1) | 78 (25.2) |
| Sex, *n* (%) |  |  |
| Female | 296 (41.3) | 129 (41.6) |
| Male | 421 (58.7) | 181 (58.4) |
| Race, *n* (%) |  |  |
| White | 698 (97.4) | 289 (93.2) |
| Non-white | 19 (2.6) | 21 (6.8) |
| Region, *n* (%) |  |  |
| North America | 227 (31.7) | 76 (24.5) |
| Europe | 389 (54.3) | 182 (58.7) |
| Australia/New Zealand/other | 101 (14.1) | 52 (16.8) |
| Baseline disease stage, *n* (%) |  |  |
| *n* | 716 | 310 |
| Unresectable IIIC, M1a, or M1b | 262 (36.6) | 120 (38.7) |
| M1c | 454 (63.4) | 190 (61.3) |
| Baseline ECOG PS, *n* (%) |  |  |
| *n* | 715 | 306 |
| 0 | 456 (63.8) | 225 (73.5) |
| 1 | 259 (36.2) | 80 (26.1) |
| 2 | 0 | 1 (0.3) |
| Baseline LDH level, *n* (%) |  |  |
| *n* | 711 | 305 |
| Normal | 400 (56.3) | 164 (53.8) |
| Elevated | 311 (43.7) | 141 (46.2) |
| Baseline liver metastases, *n* (%) |  |  |
| *n* | 713 | 310 |
| Yes | 236 (33.1) | 100 (32.3) |
| No | 477 (66.9) | 210 (67.7) |
| Baseline SLD of target lesions (mm) |  |  |
| *n* | 704 | 310 |
| Mean (SD) | 86.1 (80.1) | 74.4 (57.8) |
| Median | 68.0 | 62.5 |
| Interquartile range (Q1–Q3) | 35.5–118.3 | 33.0–98.0 |
| Range (min–max) | 9–1310 | 10–398 |

C + V = cobimetinib plus vemurafenib; ECOG PS = Eastern Cooperative Oncology Group performance status; LDH = lactate dehydrogenase; Q = quartile; SD = standard deviation; SLD = sum of longest diameters; V = vemurafenib monotherapy.

**Table S2. Time-dependent multivariate Cox proportional hazards regression modeling of survival outcomes with depth-of-response variables in pooled patient cohorts**

| **Survival Outcome** | **Pooled V Cohort**  ***n* = 717** | **Pooled C + V Cohort**  ***n* = 310** |
| --- | --- | --- |
| **PFS** |  |  |
| Event, *n* | 609 | 203 |
| K–M, median (95% CI), months | 6.9 (6.2–7.0) | 12.7 (10.6–14.7) |
| HR (95% CI)^a^ | 1.0 (reference) | 0.86 (0.72–1.03) |
| *p* value^a^ |  | 0.0945 |
| Max%SLD before/at first PD |  |  |
| HR per 1% increase in Max%SLD (95% CI)^b^ | 1.01 (1.01–1.01) | 1.01 (1.01–1.02) |
| *p* value^b^ | <0.0001 | <0.0001 |
| TimeMax%SLD before/at first PD |  |  |
| HR per 1 month increase in TimeMax%SLD (95% CI)^b^ | 0.96 (0.93–0.99) | 1.03 (0.98–1.08) |
| *p* value^b^ | 0.0033 | 0.2260 |
| **OS** |  |  |
| Event, *n* | 495 | 148 |
| K–M, median (95% CI), months | 15.0 (13.8–16.8) | 28.0 (21.8–31.2) |
| HR (95% CI)^a^ | 1.0 (reference) | 0.86 (0.70–1.06) |
| *p* value^a^ |  | 0.1563 |
| Max%SLD before/at first PD |  |  |
| HR per 1% increase in Max%SLD (95% CI)^b^ | 1.01 (1.00–1.01) | 1.01 (1.00–1.01) |
| *p* value^b^ | 0.0005 | 0.0105 |
| TimeMax%SLD before/on first PD |  |  |
| HR per 1 month increase in TimeMax%SLD (95% CI)^b^ | 0.94 (0.91–0.97) | 0.92 (0.87, 0.97) |
| *p* value^b^ | <0.0001 | 0.0042 |

C + V = cobimetinib plus vemurafenib; CI = confidence interval; HR = hazard ratio; K–M = Kaplan–Meier; Max%SLD = maximum percentage change in the sum of longest diameters; OS = overall survival; PD = disease progression; PFS = progression-free survival; SLD = sum of longest diameters; TimeMax%SLD = time to maximum percentage change in the sum of longest diameters; V = vemurafenib monotherapy.

^a^The HR and *p* values from the Cox proportional hazards regression model are for the comparison of two pooled cohorts (pooled cobimetinib plus vemurafenib versus pooled vemurafenib).

^b^The HR and *p* values from the Cox proportional hazards regression model are for Max%SLD of target lesions before or on the first PD, or TimeMax%SLD of target lesion before or at the first PD within each cohort, analysed as continuous variables within each cohort.

**Table S3. Cox proportional hazards analysis of survival outcomes with depth-of-response variables in pooled cohorts of patients with gene expression signature data**

| **Survival Outcome** | **Pooled V Cohort**  ***n* = 320** | **Pooled C + V Cohort**  ***n* = 143** |
| --- | --- | --- |
| **PFS** |  |  |
| *n* (event) | 272 | 100 |
| K–M median, months (95% CI) | 6.9 (6.1–7.2) | 11.0 (9.3–13.7) |
| HR (95% CI)^a^ | 1.0 (reference) | 0.87 (0.68–1.11) |
| *p* value^a^ |  | 0.2685 |
| Max%SLD before/on first PD |  |  |
| HR per 1% increase in Max%SLD (95% CI)^b^ | 1.02 (1.01–1.02) | 1.02 (1.02–1.03) |
| *p* value^b^ | <0.0001 | <0.0001 |
| TimeMax%SLD before/on first PD |  |  |
| HR per 1 month increase in TimeMax%SLD (95% CI)^b^ | 0.86 (0.82–0.89) | 0.80 (0.76–0.86) |
| *p* value^b^ | <0.0001 | <0.0001 |
| **OS** |  |  |
| *n* (event) | 231 | 72 |
| K–M median, months (95% CI) | 14.7 (12.6–17.4) | 24.5 (21.5–31.8) |
| HR (95% CI)^a^ | 1.0 (reference) | 0.82 (0.62–1.10) |
| *p* value^a^ |  | 0.1867 |
| Max%SLD before/on first PD |  |  |
| HR per 1% increase in Max%SLD (95% CI)^b^ | 1.01 (1.01–1.01) | 1.01 (1.00–1.02) |
| *p* value^b^ | <0.0001 | 0.0137 |
| TimeMax%SLD before/on first PD |  |  |
| HR per 1 month increase in TimeMax%SLD (95% CI)^b^ | 0.88 (0.85–0.92) | 0.86 (0.81, 0.92) |
| *p* value^b^ | <0.0001 | <0.0001 |

C + V = cobimetinib plus vemurafenib; CI = confidence interval; HR = hazard ratio; K–M = Kaplan–Meier; Max%SLD = maximum percentage change in the sum of longest diameters; OS = overall survival; PD = disease progression; PFS = progression-free survival; SLD = sum of longest diameters; TimeMax%SLD = time to maximum percentage change in the sum of longest diameters; V = vemurafenib monotherapy.

^a^The HR and *p* values from the Cox proportional hazards regression model are for the comparison of two pooled cohorts (pooled cobimetinib plus vemurafenib versus pooled vemurafenib).

^b^The HR and *p* values from the Cox proportional hazards regression model are for Max%SLD of target lesions before or on the first PD, or TimeMax%SLD of target lesion before or at the first PD within each cohort, analysed as continuous variables within each cohort.

**Table S4. Gene expression signatures identified in baseline advanced melanoma samples associated with favourable prognosis (‘immune signature’) and unfavourable prognosis (‘cell cycle’)**

| **Cell cycle genes** | **Immune signature genes** |
| --- | --- |
| *BRCA1* | *CCL5* |
| *BRIP1* | *CCND2* |
| *CCNB1* | *CCR5* |
| *CCNE1* | *CD247* |
| *FH* | *CD3E* |
| *KDM4A* | *CD4* |
| *MAP2K2* | *CD86* |
| *MTCH1* | *CD8A* |
| *MYC* | *GZMA* |
| *NF2* | *HAVCR2* |
| *PRKDC* | *IKZF1* |
| *PTK2* | *KIR3DL1* |
| *RPTOR* | *KLRK1* |
| *SMARCA4* | *LAG3* |
| *SNAI2* | *LGALS9* |
| *SOX4* | *MYD88* |
| *SRSF2* | *PDCD1LG2* |
| *WDR5* | *PIK3R5* |
| *ZNF703* | *PTGER4* |
|  | *PTPRC* |
|  | *TBX21* |
|  | *TIGIT* |
|  | *TNFRSF9* |

**Figure S1.** Landmark PFS and OS rates by tumour reduction quartiles based on the vemurafenib monotherapy cohort (**A** and **C**), or based on the cobimetinib plus vemurafenib cohort (**B** and **D**).

**
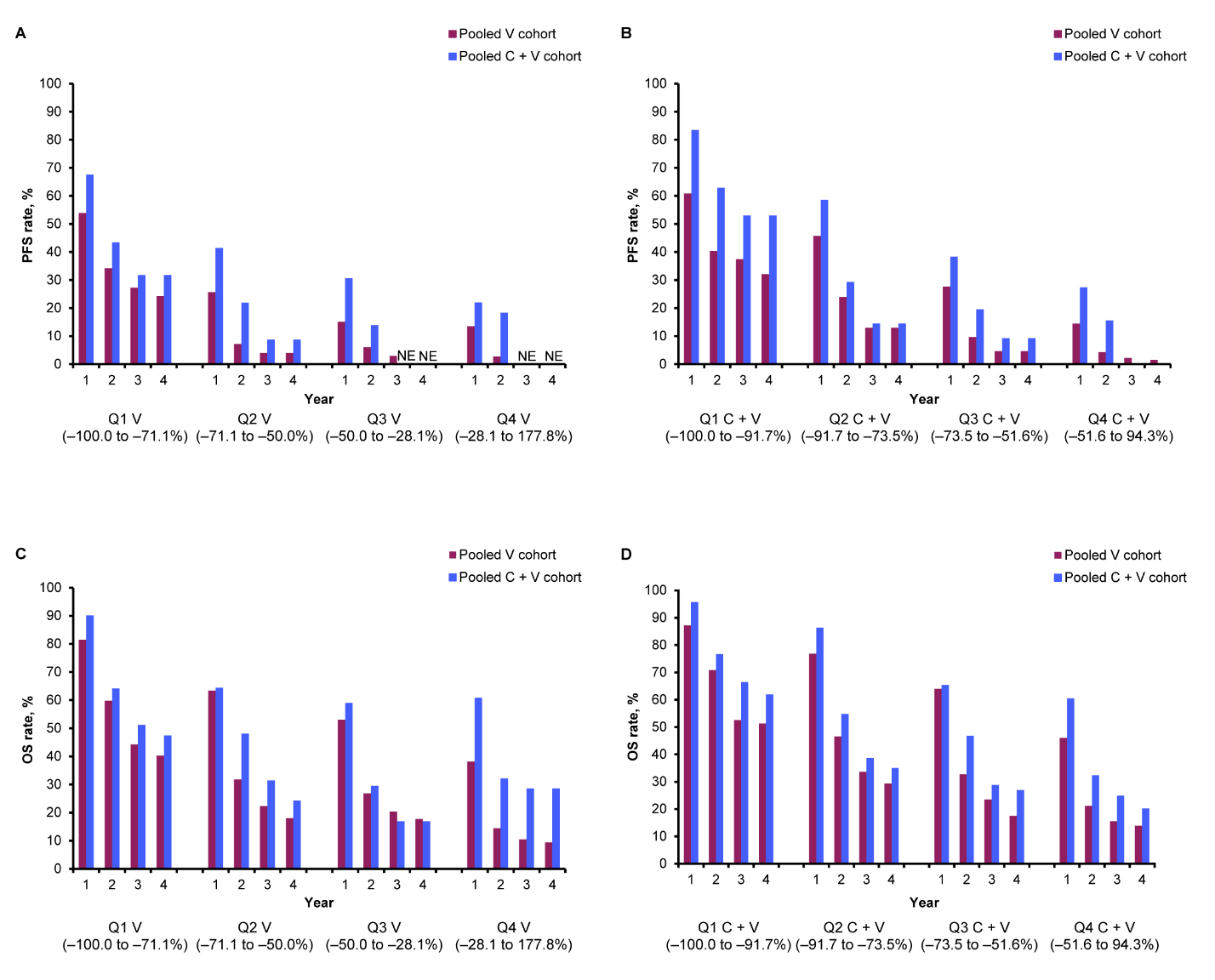
**C + V, cobimetinib plus vemurafenib; NE, not estimable; PFS, progression-free survival;
Q, quartile; V, vemurafenib monotherapy.
