# Supplementary material for: Impact of depth of response on survival in patients treated with cobimetinib ± vemurafenib: pooled analysis of BRIM-2, BRIM-3, BRIM-7 and coBRIM
Source: Br J Cancer. 2019 Aug 16;121(7):522–8. doi: 10.1038/s41416-019-0546-y (PMC6889491; doi:10.1038/s41416-019-0546-y)
Supplement: Supplementary file 2 — Supplemental IRB list [file 41416_2019_546_MOESM2_ESM.pdf]

BRIM2 IRB List

Study NP22657 IRB / EC List

IND: 73,620

Institution	Site No.	CRTN	IRB / IEC Name	Address	Action(s)	Approval Dates
University of Colorado	102	165862	Western Institutional Review Board	3535 Seventh Ave, SW Olympia, WA 98502	Initial Approval<br>ICF (17 yr old)<br>Protocol B<br>ICF	16-Oct-09<br>20-Nov-09<br>28-May-10<br>10-Sep-10
Calvary Mates Newcastle, AUS	103	165550	Hunter New England Human Research Ethics Committee	Locked Bag 1<br>New Lambton, NSW 2305<br>AUSTRALIA	Initial Approval<br>ICF<br>Protocol B	13-Aug-09<br>25-Mar-10<br>15-Jul-10
TX Oncology-Baylor Cancer Center	104	165664	Copernicus Group IRB	One Triangle Drive<br>Suite 100<br>P.O. Box 110605<br>Research Triangle Park, NC 27709	Initial Approval<br>ICF, IB Adden 2<br>Pt. Eligibility Checklist<br>IB Adden 3<br>Protocol B, ICF<br>Re-Approval of Study	3-Sep-09<br>2-Oct-09<br>19-Nov-09<br>11-Dec-09<br>31-Mar-10<br>24-Jun-10
Westmeade Hospital, AUS	105	166171	Hunter New England Human Research Ethics Committee	Locked Bag 1<br>New Lambton, NSW 2305<br>AUSTRALIA	ICF ver. 2 & 3<br>Initial Approval<br>ICF<br>Protocol B	25-Mar-10<br>13-Aug-09<br>25-Mar-10<br>15-Jul-10

BRIM2 IRB List

Institution	Site No.	CRTN	IRB / IEC Name	Address	Action(s)	Approval Dates
MD Anderson	106	165728	The University of Texas MD Anderson Cancer Center Institutional Review Board	Surveillance Committee<br>1515 Holcombe Blvd., Unit 083<br>Houston, TX 77030	Initial Approval<br>ICF<br>IB 5, Addendum 3<br>Protocol B	27-Aug-09<br>12-Nov-09<br>15-Jan-10<br><br>10-Jun-10
Hilman Cancer Center	107	165729	Copernicus Group IRB	One Triangle Drive<br>Suite 100<br>P.O. Box 110605<br>Research Triangle Park, NC 27709	Initial Approval<br><br>IB Adden 3<br>Protocol B, ICF<br>Re-Approval of Study	19-Nov-09<br><br>11-Dec-09<br>31-Mar-10<br>24-Jun-10
Massachusetts General	108	165727	Dana-Farber/Partners CancerCare	Office for Human Research Studies<br>Dana Farber Cancer Institute<br>20 Overland Street, 2nd Floor<br>Boston, MA 02115	Initial Approval<br>Protocol B<br>IB 6	15-Sep-09<br>20-May-10<br>17-Mar-10
Peter MacCallum Cancer Ctr, AUS	109	165860	Peter MacCallum Cancer Centre Human Research Ethics Committee	Peter MacCallum Cancer Centre<br>St. Andrews Place<br>East Melbourne 3002<br>Victoria Australia	Initial Approval<br>ICF; IB's<br>IB 6<br>Protocol B	29-Sep-09<br>17-Dec-09<br>24-May-10<br>30-Jul-10

BRIM2 IRB List

Institution	Site No.	CRTN	IRB / IEC Name	Address	Action(s)	Approval Dates
New York University Medical Center	114	165998	NYU School of Medicine IRB	550 First Avenue<br>New York, NY 10016	Initial Approval<br>IB 5, Addendum 2 & 3<br>IB 6<br>Protocol B, ICF	30-Oct-09<br>11-Dec-09<br><br>01-Mar-10<br>27-Apr-10
Vanderbilt	110	165551	Vanderbilt University IRB	1313 21st Avenue South<br>504 Oxford House<br>Nashville, TN 37232	Initial Approval<br>ICF, 1572<br>Protocol B, ICF, IB 6<br>Pt. Letter; ICF	2-Nov-09<br>20-Jan-10<br>26-Apr-10<br><br>26-Aug-10
UCLA	111	166172	UCLA IRB	Office for the Protection of Research Subjects<br>11000 Kinross Avenue<br>Los Angeles, CA 90095	Initial Approval<br>Patient Diary<br>IB adden 2 & 3, ICF<br>Protocol B, ICF<br>Continuing Review	11-Sep-09<br>07-Oct-09<br>4-Nov-09<br><br>21-Apr-10<br>23-Aug-10

BRIM2 IRB List

Institution	Site No.	CRTN	IRB / IEC Name	Address	Action(s)	Approval Dates
University of Pennsylvania	112	165553	University of Pennsylvania IRB	Committee on Studies Involving Human Beings<br>Office of Regulatory Affairs<br>University of Pennsylvania<br>3624 Market Street<br>Suite 301S<br>Philadelphia, PA 19104	Initial Approval<br>ICF<br>ICF; IB's<br>ICF<br>Continuing Review<br>ICF w/ HIPAA<br>Protocol B, ICF, IB 6	25-Aug-09<br>10-Sep-09<br>06-Jan-10<br>17-Feb-10<br>12-Aug-10<br>15-Dec-09<br>13-May-10
Moffitt Cancer Center	113	165730	Quorum Review	Quorum Review, Inc<br>1601 Fifth Ave. Ste 1000<br>Seattle, WA 98101	Initial Approval<br>Protocol B, ICF<br>Protocol Clarification Ltr<br>Re-Approval	28-Aug-09<br>19-Apr-10<br>10-Jun-10<br>13-Aug-10
Dana Farber	116	167097	Dana-Farber/Partners CancerCare	Office for Human Research Studies<br>Dana Farber Cancer Institute<br>20 Overland Street, 2nd Floor<br>Boston, MA 02115	Activation	06-Nov-09

BRIM2 IRB List

Institution	Site No.	CRTN	IRB / IEC Name	Address	Action(s)	Approval Dates
Beth Israel	115	167098	Dana-Farber/Partners CancerCare	Office for Human Research Studies<br>Dana Farber Cancer Institute<br>20 Overland Street, 2nd Floor<br>Boston, MA 02115	Activation	30-Oct-09

BRIM3 IRB List

CRTN	INVESTIGATOR	IRB/IEC Name	IRB/IEC Address	Country	Approval Dates	Date
201214	Michael Millward	Sir Charles Gairdner Group Human Research Ethics Committee	1st Floor E Block, Sir Charles Gairdner Hospital<br>Hospital Avenue<br>Nedlands, Western Australia 6009	Australia	Protocol Version A<br>Amendment B<br>Amendment C	11-FEB-10<br>15-SEP-10<br>01-DEC-10
201001	Phillip Parente	Eastern Health Research & Ethics Committee	5 Arnold St<br>Box Hill, Victoria 3128	Australia	Protocol Version A<br>Amendment B<br>Amendment C	29-JAN-10<br>08-SEP-10<br>15-DEC-10
201244	Min-Ne Wu	Bellberry Human Research Ethics Committee	Bellberry Limited<br>229 Greenhill Road<br>Dulwich, South Australia 5065	Australia	Protocol Version A<br>Amendment B<br>Amendment C	27-NOV-09<br>09-JUL-10<br>19-NOV-10
201009	Felix Couture	Comité d'éthique de l'hôtel Dieu de Quebec	11, cote du Palais<br>Quebec, Quebec<br>G1R 2J6	Canada	Protocol Version A<br>Amendment B<br>Amendment C	23-APR-10<br>14-DEC-10<br>18-JAN-11
201217	David Hogg	Ontario Cancer REB	101 College St, Ste 800<br>Toronto, Ontario<br>M5G 0A3	Canada	Protocol Version A<br>Amendment B<br>Amendment C	25-MAR-10<br>23-DEC-10<br>19-JAN-11
201004	Elaine McWhirter	Ontario Cancer REB	MaRS Centre, South Tower<br>101 College St, Ste 500<br>Toronto, Ontario M5G 1L7	Canada	Protocol Version A<br>Amendment B<br>Amendment C	30-APR-10<br>Approval pending as of 04-MAR-11 for B and C
201005	Wilson Miller	McGill Faculty of Medicine REB	3655 Promenade Sir William Osler # 633<br>Montreal, Quebec<br>H3G 1Y6	Canada	Protocol Version A<br>Amendment B<br>Amendment C	24-FEB-10<br>25-OCT-10<br>02-DEC-10
203103	Wilson Miller	McGill Faculty of Medicine REB	3655 Promenade Sir William Osler # 633<br>Montreal, Quebec<br>H3G 1Y6	Canada	Protocol Version A<br>Amendment B<br>Amendment C	24-FEB-10<br>25-OCT-10<br>02-DEC-10

BRIM3 IRB List

CRTN	INVESTIGATOR	IRB/IEC Name	IRB/IEC Address	Country	Approval Dates	Date
201003	Teresa Petrella	Ontario Cancer REB	101 College Street, Suite 800<br>Toronto, Ontario M5G 0A3	Canada	Protocol Version A<br>Amendment B<br>Amendment C	09-JUN-10<br>Approval pending as of 04-MAR-11 for B and C
201008	Michael Smylie	Alberta Cancer REC	1500-10123 99 Street<br>Edmonton, Alberta<br>T5J 3H1	Canada	Protocol Version A<br>Amendment B<br>Amendment C	22-MAR-10<br>15-OCT-10<br>10-FEB-11
201010	Ralph Wong	Bannatyne Campus REB	770 Bannatyne Ave<br>Winnipeg, Manatoba R3E 0W3	Canada	Protocol Version A<br>Amendment B<br>Amendment C	21-APR-10<br>10-NOV-10<br>As per REB, no need for submission and approval as it has no effect on patient care for this site
201183	Didier Cupissol	CPP IDF III	Hôpital Tarnier-Cochin<br>89 rue d'Assas<br>Paris 75006	France	Protocol Version A<br>Amendment B<br>Amendment C	11-JAN-10<br>20-SEP-10<br>01-FEB-11
201193	Brigitte Dreno	CPP IDF III	Hôpital Tarnier-Cochin<br>89 rue d'Assas<br>Paris 75006	France	Protocol Version A<br>Amendment B<br>Amendment C	11-JAN-10<br>20-SEP-10<br>01-FEB-11
202616	Jean Jacques Grob	CPP IDF III	Hôpital Tarnier-Cochin<br>89 rue d'Assas<br>Paris 75006	France	Protocol Version A<br>Amendment B<br>Amendment C	15-MAR-10<br>20-SEP-10<br>01-FEB-10

BRIM3 IRB List

Names and Addresses of Institutional Review Boards / Ethics Committees and Actions

Protocol: NO25026

CRTN	INVESTIGATOR	IRB/IEC Name	IRB/IEC Address	Country	Approval Dates	Date
200978	Victoria Atkinson	Princess Alexandra Hospital Human Research Ethics Committee	Level 2, Building 35<br>Princess Alexandra Hospital Ipswich Road<br>Woolloongabba, Queensland 4102	Australia	Protocol Version A<br>Amendment B<br>Amendment C	04-MAR-10<br>07-SEP-10<br>08-DEC-10
200981	Fran Boyle	Hunter New England Research Ethics Committee	Lookout Road<br>New Lambton, New South Wales 2305	Australia	Protocol Version A<br>Amendment B<br>Amendment C	09-DEC-09<br>29-SEP-10<br>02-DEC-10
201049	Benjamin Brady	Cabrini Human Research Ethics Committee	Cabrini Institute<br>183 Wattletree Road<br>Malvern, Victoria 3144	Australia	Protocol Version A<br>Amendment B<br>Amendment C	22-FEB-10<br>10-SEP-10<br>02-DEC-10
201213	Melissa Eastgate	RBWH Human Research Ethics Committee (HREC)	Level 7, Block 7<br>Royal Brisbane and Women's Hospital<br>Brisbane, Queensland 4006	Australia	Protocol Version A<br>Amendment B<br>Amendment C	03-MAR-10<br>05-AUG-10<br>30-NOV-10
201051	Alexander Guminski	Hunter New England Research Ethics Committee	Lookour Rd<br>New Lambton, New South Wales 2305	Australia	Protocol Version A<br>Amendment B<br>Amendment C	09-DEC-09<br>29-SEP-10<br>02-DEC-10
201052	Peter Hersey	Hunter New England Research Ethics Committee	Lookout Road<br>New Lambton, New South Wales 2305	Australia	Protocol Version A<br>Amendment B<br>Amendment C	09-DEC-09<br>29-SEP-10<br>02-DEC-10
200990	Rick Kefford	Hunter New England Research Ethics Committee	Lookout Road<br>New Lambton, New South Wales 2305	Australia	Protocol Version A<br>Amendment B<br>Amendment C	09-DEC-09<br>29-SEP-10<br>02-DEC-10
201247	Grant McArthur	Peter MacCallum Cancer Centre Human Research Ethics Committee	Peter MacCallum Cancer Centre<br>St. Andrews Place<br>East Melbourne, Victoria 3002	Australia	Protocol Version A<br>Amendment B<br>Amendment C	18-FEB-10<br>04-AUG-10<br>02-DEC-10

BRIM3 IRB List

CRTN	INVESTIGATOR	IRB/IEC Name	IRB/IEC Address	Country	Approval Dates	Date
201059	Pascal Joly	CPP IDF III	Hôpital Tarnier-Cochin<br>89 rue d'Assas<br>Paris 75006	France	Protocol Version A<br>Amendment B<br>Amendment C	11-JAN-10<br>20-SEP-10<br>01-FEB-11
201060	Thomas Jouary	CPP IDF III	Hôpital Tarnier-Cochin<br>89 rue d'Assas<br>Paris 75006	France	Protocol Version A<br>Amendment B<br>Amendment C	11-JAN-10<br>20-SEP-10<br>01-FEB-11
201061	Celeste Lebbe	CPP IDF III	Hôpital Tarnier-Cochin<br>89 rue d'Assas<br>Paris 75006	France	Protocol Version A<br>Amendment B<br>Amendment C	11-JAN-10<br>20-SEP-10<br>01-FEB-11
201185	Laurent Mortier	CPP IDF III	Hôpital Tarnier-Cochin<br>89 rue d'Assas<br>Paris 75006	France	Protocol Version A<br>Amendment B<br>Amendment C	11-JAN-10<br>20-SEP-10<br>01-FEB-11
201194	Jean Paul Ortonne	CPP IDF III	Hôpital Tarnier-Cochin<br>89 rue d'Assas<br>Paris 75006	France	Protocol Version A<br>Amendment B<br>Amendment C	11-JAN-10<br>20-SEP-10<br>01-FEB-11
201186	Caroline Robert	CPP IDF III	Hôpital Tarnier-Cochin<br>89 rue d'Assas<br>Paris 75006	France	Protocol Version A<br>Amendment B<br>Amendment C	11-JAN-10<br>20-SEP-10<br>01-FEB-11
201187	Luc Thomas	CPP IDF III	Hôpital Tarnier-Cochin<br>89 rue d'Assas<br>Paris 75006	France	Protocol Version A<br>Amendment B<br>Amendment C	11-JAN-10<br>20-SEP-10<br>01-FEB-11
201197	Carola Berking	EC Med. Faculty CA University	Universitaetsklinikum<br>Schwanenweg 20<br>Kiel, Schleswig Holstein 24105	Germany	Protocol Version A<br>Amendment B<br>Amendment C	17-FEB-10<br>12-AUG-10<br>14-FEB-11
201198	Alexander Enk	EC Med. Faculty CA University	Universitaetsklinikum<br>Schwanenweg 20<br>Kiel, Schleswig Holstein 24105	Germany	Protocol Version A<br>Amendment B<br>Amendment C	17-FEB-10<br>12-AUG-10<br>14-FEB-11

BRIM3 IRB List

CRTN	INVESTIGATOR	IRB/IEC Name	IRB/IEC Address	Country	Approval Dates	Date
201201	Claus Garbe	EC Med. Faculty CA University	Universitaetsklinikum<br>Schwanenweg 20<br>Kiel, Schleswig Holstein 24105	Germany	Protocol Version A<br>Amendment B<br>Amendment C	17-FEB-10<br>12-AUG-10<br>14-FEB-11
201196	Anja Gesierich	EC Med. Faculty CA University	Universitaetsklinikum<br>Schwanenweg 20<br>Kiel, Schleswig Holstein 24105	Germany	Protocol Version A<br>Amendment B<br>Amendment C	17-MAY-10<br>12-AUG-10<br>14-FEB-11
201064	Ralf Gutzmer	EC Med. Faculty CA University	Universitaetsklinikum<br>Schwanenweg 20<br>Kiel, Schleswig Holstein 24105	Germany	Protocol Version A<br>Amendment B<br>Amendment C	17-FEB-10<br>12-AUG-10<br>14-FEB-11
203360	Christian Hafner	EC Med. Faculty CA University	Universitaetsklinikum<br>Schwanenweg 20<br>Kiel, Schleswig Holstein 24105	Germany	Protocol Version A<br>Amendment B<br>Amendment C	25-MAY-10<br>12-AUG-10<br>14-FEB-11
201065	Axel Hauschild	EC Med. Faculty CA University	Universitaetsklinikum<br>Schwanenweg 20<br>Kiel, Schleswig Holstein 24105	Germany	Protocol Version A<br>Amendment B<br>Amendment C	17-FEB-10<br>12-AUG-10<br>14-FEB-11
203151	Rudolf Herbst	EC Med. Faculty CA University	Universitaetsklinikum<br>Schwanenweg 20<br>Kiel, Schleswig Holstein 24105	Germany	Protocol Version A<br>Amendment B<br>Amendment C	25-MAY-10<br>12-AUG-10<br>14-FEB-11
201199	Sabine Sell	EC Med. Faculty CA University	Universitaetsklinikum<br>Schwanenweg 20<br>Kiel, Schleswig Holstein 24105	Germany	Protocol Version A<br>Amendment B<br>Amendment C	17-FEB-10<br>12-AUG-10<br>14-FEB-11
201200	Roland Kaufmann	EC Med. Faculty CA University	Universitaetsklinikum<br>Schwanenweg 20<br>Kiel, Schleswig Holstein 24105	Germany	Protocol Version A<br>Amendment B<br>Amendment C	17-FEB-10<br>12-AUG-10<br>14-FEB-11
201202	Carmen Loquai	EC Med. Faculty CA University	Universitaetsklinikum<br>Schwanenweg 20<br>Kiel, Schleswig Holstein 24105	Germany	Protocol Version A<br>Amendment B<br>Amendment C	17-FEB-10<br>12-AUG-10<br>14-FEB-11

BRIM3 IRB List

CRTN	INVESTIGATOR	IRB/IEC Name	IRB/IEC Address	Country	Approval Dates	Date
201203	Cornelia Mauch	EC Med. Faculty CA University	Universitaetsklinikum<br>Schwanenweg 20<br>Kiel, Schleswig Holstein 24105	Germany	Protocol Version A<br>Amendment B<br>Amendment C	17-FEB-10<br>12-AUG-10<br>14-FEB-11
201066	Peter Mohr	EC Med. Faculty CA University	Universitaetsklinikum<br>Schwanenweg 20<br>Kiel, Schleswig Holstein 24105	Germany	Protocol Version A<br>Amendment B<br>Amendment C	17-FEB-10<br>12-AUG-10<br>14-FEB-11
201204	Dirk Schadendorf	EC Med. Faculty CA University	Universitaetsklinikum<br>Schwanenweg 20<br>Kiel, Schleswig Holstein 24105	Germany	Protocol Version A<br>Amendment B<br>Amendment C	17-FEB-10<br>12-AUG-10<br>14-FEB-11
201207	Jan Simon	EC Med. Faculty CA University	Universitaetsklinikum<br>Schwanenweg 20<br>Kiel, Schleswig Holstein 24105	Germany	Protocol Version A<br>Amendment B<br>Amendment C	17-FEB-10<br>12-AUG-10<br>14-FEB-11
201205	Rudolf Stadler	EC Med. Faculty CA University	Universitaetsklinikum<br>Schwanenweg 20<br>Kiel, Schleswig Holstein 24105	Germany	Protocol Version A<br>Amendment B<br>Amendment C	17-FEB-10<br>12-AUG-10<br>14-FEB-11
201206	Annette Stein	EC Med. Faculty CA University	Universitaetsklinikum<br>Schwanenweg 20<br>Kiel, Schleswig Holstein 24105	Germany	Protocol Version A<br>Amendment B<br>Amendment C	17-FEB-10<br>12-AUG-10<br>14-FEB-11
201218	Michal Lotem	Hadassah Medical Organization Helsinki Committee	Kiryat Hoadassah, p.o.b. 12000<br>Jerusalem 91120	Israel	Protocol Version A<br>Amendment B<br>Amendment C	27-DEC-09<br>14-SEP-10<br>11-JAN-11
201056	Ilan Ron	Tel Aviv Sourasky Mecidal Center Helsinki Committee	6, Weizman st<br>Tel Aviv 64239	Israel	Protocol Version A<br>Amendment B<br>Amendment C	21-MAR-10<br>09-AUG-10<br>06-JAN-11
201219	Jacob Schachter	Chaim Sheba helsinki Committee	Oncology Division<br>Chaim Sehba Medical Center<br>Tel Hashomer, Ramat Gan, 52621	Israel	Protocol Version A<br>Amendment B<br>Amendment C	25-FEB-10<br>28-JUL-10<br>30-NOV-10

BRIM3 IRB List

vemurafenib (RO5185426) Roche Clinical Study Report - Protocol NO25026 - Research Report 1039652   2628

CRTN	INVESTIGATOR	IRB/IEC Name	IRB/IEC Address	Country	Approval Dates	Date
201188	Paolo Ascierto	EC IRCCS STUDIO E CURA DEI TUMORI GIOVANNI PASCALE	VIA MARIANO SEMMOLA<br>Napoli 80131	Italy	Protocol Version A<br>Amendment B<br>Amendment C	04-FEB-10<br>29-SEP-10<br>Approval not received yet
201062	Michele Del Vecchio	EC IRCCS ISTITUTO NAZIONALE TUMORI	Via Giacomo Venezian, 1<br>Milano 20133	Italy	Protocol Version A<br>Amendment B<br>Amendment C	23-FEB-10<br>25-NOV-10<br>Approval pending as of 04-MAR-11
204616	Francesco Cognetti	EC IFO – Istituti Fisioterapici Ospitalieri	Via Elio Chianesi, 53<br>Roma 00144	Italy	Protocol Version A<br>Amendment B<br>Amendment C	19-JUL-10<br>19-JUL-10<br>17-JAN-11
201190	Michele Guida	EC IRCCS OSPEDALE ONCOLOGICO DI BARI	Via S.F. Hahnemann , 10<br>Bari 70126	Italy	Protocol Version A<br>Amendment B<br>Amendment C	21-MAR-10<br>22-SEP-10<br>11-JAN-11
201191	Michele Maio	EC AOU SENESE DI SIENA	c/o U.O.C. Farmacia AOUS - Viale Bracci<br>Siena 53100	Italy	Protocol Version A<br>Amendment B<br>Amendment C	21-DEC-09<br>15-SEP-10<br>16-DEC-10
201063	Paola Queirolo	EC ISITUTO NAZIONALE RICERCA SUL CANCRO DI GENOVA	L.go R. BENZI 10<br>Genova 16132	Italy	Protocol Version A<br>Amendment B<br>Amendment C	29-DEC-09<br>05-JUL-10<br>22-NOV-10
202892	Salvatore Siena	EC Ospedale Niguarda Ca'Granda	P.zza Ospedale Maggiore 3<br>Milano 20162	Italy	Protocol Version A<br>Amendment B<br>Amendment C	22-APR-10<br>16-SEP-10<br>28-JAN-11
201192	Alessandro Testori	EC ISTITUTO EUROPEO DI ONCOLOGIA	via Ripamonti 435<br>Milano 20141	Italy	Protocol Version A<br>Amendment B<br>Amendment C	09-MAR-10<br>24-SEP-10<br>04-MAR-11

BRIM3 IRB List

CRTN	INVESTIGATOR	IRB/IEC Name	IRB/IEC Address	Country	Approval Dates	Date
201233	J.B.A.G. Haanen	NKI Protocool Toetsingscommissie (Central)	Plesmanlaan 121<br>Amsterdam 1066 CX	Netherlands	Protocol Version A<br>Amendment B<br>Amendment C	08-FEB-10<br>16-AUG-10<br>26-JAN-11
202388	G.A.P. Hospers	NKI Protocool Toetsingscommissie (Central)	Plesmanlaan 121<br>Amsterdam 1066 CX	Netherlands	Protocol Version A<br>Amendment B<br>Amendment C	08-FEB-10<br>16-AUG-10<br>26-JAN-11
201165	A.J.M. Van den Eertwegh	NKI Protocool Toetsingscommissie (Central)	Plesmanlaan 121<br>Amsterdam 1066 CX	Netherlands	Protocol Version A<br>Amendment B<br>Amendment C	08-FEB-10<br>16-AUG-10<br>26-JAN-11
201246	Catherine Barrow	Multi- Region EC Committee	C/O Ministry of Health<br>Wellington	New Zealand	Protocol Version A<br>Amendment B<br>Amendment C	24-MAR-10<br>01-SEP-10<br>10-DEC-10
201054	Richard Isaacs	Multi- Region EC Committee	C/O Ministry of Health<br>Wellington	New Zealand	Protocol Version A<br>Amendment B<br>Amendment C	24-MAR-10<br>01-SEP-10<br>10-DEC-10
201215	Chris Jackson	Multi- Region EC Committee	C/O Ministry of Health<br>Wellington	New Zealand	Protocol Version A<br>Amendment B<br>Amendment C	24-MAR-10<br>01-SEP-10<br>10-DEC-10
201055	Michael Jameson	Multi- Region EC Committee	C/O Ministry of Health<br>Wellington	New Zealand	Protocol Version A<br>Amendment B<br>Amendment C	24-MAR-10<br>01-SEP-10<br>10-DEC-10
201216	Mike McCrystal	Multi- Region EC Committee	C/O Ministry of Health<br>Wellington	New Zealand	Protocol Version A<br>Amendment B<br>Amendment C	24-MAR-10<br>01-SEP-10<br>10-DEC-10
201235	Johan Hansson	Regionala Etikprövningsnämnden i Stockholm	FE 289<br>Stockholm SE-171 77	Sweden	Protocol Version A<br>Amendment B<br>Amendment C	20-JAN-10<br>14-SEP-10<br>16-NOV-10

BRIM3 IRB List

CRTN	INVESTIGATOR	IRB/IEC Name	IRB/IEC Address	Country	Approval Dates	Date
201236	Ingrid Ljuslinder	Regionala Etikprövningsnämnden i Stockholm	FE 289<br>Stockholm SE-171 77	Sweden	Protocol Version A<br>Amendment B<br>Amendment C	20-JAN-10<br>14-SEP-10<br>16-NOV-10
201250	Lotta Lundgren	Regionala Etikprövningsnämnden i Stockholm	FE 289<br>Stockholm SE-171 77	Sweden	Protocol Version A<br>Amendment B<br>Amendment C	20-JAN-10<br>14-SEP-10<br>16-NOV-10
201166	Gunnar Wagenius	Regionala Etikprövningsnämnden i Stockholm	FE 289<br>Stockholm SE-171 77	Sweden	Protocol Version A<br>Amendment B<br>Amendment C	20-JAN-10<br>14-SEP-10<br>16-NOV-10
201208	Thomas Walz	Regionala Etikprövningsnämnden i Stockholm	FE 289<br>Stockholm SE-171 77	Sweden	Protocol Version A<br>Amendment B<br>Amendment C	20-JAN-10<br>14-SEP-10<br>16-NOV-10
201249	Reinhard Dummer	KEK Zürich	Sonneggstrasse 12<br>Zürich 8091	Switzerland	Protocol Version A<br>Amendment B<br>Amendment C	25-JAN-10<br>22-OCT-10<br>16-NOV-10
201220	Olivier Michielin	EC Vaud	rue du Bugnon 21<br>Lausanne 1011	Switzerland	Protocol Version A<br>Amendment B<br>Amendment C	12-APR-10<br>27-AUG-10<br>14-JAN-11
201238	Ewan Brown	South East London REC 2	Ethics Committee Office<br>Governor's Hall Suite, St Thomas'<br>Hospital<br>London SE1 7EH	United Kingdom	Protocol Version A<br>Amendment B<br>Amendment C	18-NOV-09<br>18-AUG-10<br>15-DEC-10
201209	David Chao	South East London REC 2	Ethics Committee Office<br>Governor's Hall Suite, St Thomas'<br>Hospital<br>London SE1 7EH	United Kingdom	Protocol Version A<br>Amendment B<br>Amendment C	18-NOV-09<br>18-AUG-10<br>15-DEC-10
201210	Pippa Corrie	South East London REC 2	Ethics Committee Office<br>Governor's Hall Suite, St Thomas'<br>Hospital<br>London SE1 7EH	United Kingdom	Protocol Version A<br>Amendment B<br>Amendment C	18-NOV-09<br>18-AUG-10<br>15-DEC-10

BRIM3 IRB List

CRTN	INVESTIGATOR	IRB/IEC Name	IRB/IEC Address	Country	Approval Dates	Date
201232	Jeff Evans	South East London REC 2	Ethics Committee Office<br>Governor's Hall Suite, St Thomas'<br>Hospital<br>London SE1 7EH	United Kingdom	Protocol Version A<br>Amendment B<br>Amendment C	18-NOV-09<br>18-AUG-10<br>15-DEC-10
201237	Mark Harries	South East London REC 2	Ethics Committee Office<br>Governor's Hall Suite, St Thomas'<br>Hospital<br>London SE1 7EH	United Kingdom	Protocol Version A<br>Amendment B<br>Amendment C	18-NOV-09<br>18-AUG-10<br>15-DEC-10
202938	James Larkin	South East London REC 2	Ethics Committee Office<br>Governor's Hall Suite, St Thomas'<br>Hospital<br>London SE1 7EH	United Kingdom	Protocol Version A<br>Amendment B<br>Amendment C	18-NOV-09<br>18-AUG-10<br>15-DEC-10
201239	James Larkin	South East London REC 2	Ethics Committee Office<br>Governor's Hall Suite, St Thomas'<br>Hospital<br>London SE1 7EH	United Kingdom	Protocol Version A<br>Amendment B<br>Amendment C	18-NOV-09<br>18-AUG-10<br>15-DEC-10
201240	Paul Lorigan	South East London REC 2	Ethics Committee Office<br>Governor's Hall Suite, St Thomas'<br>Hospital<br>London SE1 7EH	United Kingdom	Protocol Version A<br>Amendment B<br>Amendment C	18-NOV-09<br>18-AUG-10<br>15-DEC-10
201211	Mark Middleton	South East London REC 2	Ethics Committee Office<br>Governor's Hall Suite, St Thomas'<br>Hospital<br>London SE1 7EH	United Kingdom	Protocol Version A<br>Amendment B<br>Amendment C	18-NOV-09<br>18-AUG-10<br>15-DEC-10
201212	Paul Nathan	South East London REC 2	Ethics Committee Office<br>Governor's Hall Suite, St Thomas'<br>Hospital<br>London SE1 7EH	United Kingdom	Protocol Version A<br>Amendment B<br>Amendment C	18-NOV-09<br>18-AUG-10<br>15-DEC-10
201241	Christian Ottensmeier	South East London REC 2	Ethics Committee Office<br>Governor's Hall Suite, St Thomas'<br>Hospital<br>London SE1 7EH	United Kingdom	Protocol Version A<br>Amendment B<br>Amendment C	18-NOV-09<br>18-AUG-10<br>15-DEC-10

vemurafenib (RO5185426) Roche Clinical Study Report - Protocol NO25026 -
Research Report 1039652   2631

BRIM3 IRB List

CRTN	INVESTIGATOR	IRB/IEC Name	IRB/IEC Address	Country	Approval Dates	Date
201242	Poulam Patel	South East London REC 2	Ethics Committee Office<br>Governor's Hall Suite, St Thomas' Hospital<br>London SE1 7EH	United Kingdom	Protocol Version A<br>Amendment B<br>Amendment C	18-NOV-09<br>18-AUG-10<br>15-DEC-10
203236	Ruth Plummer	South East London REC 2	Ethics Committee Office<br>Governor's Hall Suite, St Thomas' Hospital<br>London SE1 7EH	United Kingdom	Protocol Version A<br>Amendment B<br>Amendment C	18-NOV-09<br>18-AUG-10<br>15-DEC-10
203183	John Wagstaff	South East London REC 2	Ethics Committee Office<br>Governor's Hall Suite, St Thomas' Hospital<br>London SE1 7EH	United Kingdom	Protocol Version A<br>Amendment B<br>Amendment C	18-NOV-09<br>18-AUG-10<br>15-DEC-10
202384	Virginia Wolstenholme	South East London REC 2	Ethics Committee Office<br>Governor's Hall Suite, St Thomas' Hospital<br>London SE1 7EH	United Kingdom	Protocol Version A<br>Amendment B<br>Amendment C	18-NOV-09<br>18-AUG-10<br>15-DEC-10
203149	Wallace Akerley	Huntsman Cancer Institute at University of Utah - IRB	75 South 2000 East, # 111<br>Salt Lake City, UT. 84112	USA	Protocol Version A<br>Amendment B<br>Amendment C	12-MAY-10<br>13-OCT-10<br>09-FEB-11
201156	Paul Chapman	Memorial Sloan-Kettering Cancer Center IRB/Privacy Board	1275 York Avenue<br>New York, NY. 10021	USA	Protocol Version A<br>Amendment B<br>Amendment C	26-JAN-10<br>11-AUG-10<br>22-FEB-11
200992	Fran Collichio	The University of North Carolina at Chapel Hill<br>Office of Human Research Ethics	Medical School Building 52<br>Mason Farm Road<br>CB #7097<br>Chapel Hill, NC. 27599-7097	USA	Protocol Version A<br>Amendment B<br>Amendment C	01-MAR-10<br>13-AUG-10<br>07-JAN-11
202618	Robert Conry	WIRB	3535 Seventh Ave, SW<br>Olympia, WA. 98508	USA	Protocol Version A<br>Amendment B<br>Amendment C	30-MAR-10<br>18-AUG-10<br>21-DEC-10

BRIM3 IRB List

CRTN	INVESTIGATOR	IRB/IEC Name	IRB/IEC Address	Country	Approval Dates	Date
200993	Lee Cranmer	WIRB	3535 Seventh Ave, SW<br>Olympia, WA. 98508	USA	Protocol Version A<br>Amendment B<br>Amendment C	20-APR-10<br>24-AUG-10<br>20-DEC-10
201000	Brendan Curti	Providence Health & Services Institutional Review Board	5251 NE Glisan St<br>Building A – 3rd Floor<br>Portland, OR. 97213	USA	Protocol Version A<br>Amendment B<br>Amendment C	06-MAY-10<br>28-SEP-10<br>24-JAN-11
202622	Keith Flaherty (Beth Israel)	Office for Human Research Studies	Dana Farber Cancer Institute<br>20 Overland St, 2nd Floor<br>Boston, MA. 02115	USA	Protocol Version A<br>Amendment B<br>Amendment C	11-JAN-10<br>29-NOV-10<br>22-FEB-11
202623	Keith Flaherty (Dana Farber)	Office for Human Research Studies	Dana Farber Cancer Institute<br>20 Overland St, 2nd Floor<br>Boston, MA. 02115	USA	Protocol Version A<br>Amendment B<br>Amendment C	11-JAN-10<br>29-NOV-10<br>22-FEB-11
200968	Keith Flaherty (Mass Gen)	Office for Human Research Studies	Dana Farber Cancer Institute<br>20 Overland St, 2nd Floor<br>Boston, MA. 02115	USA	Protocol Version A<br>Amendment B<br>Amendment C	11-JAN-10<br>29-NOV-10<br>22-FEB-11
200994	Lawrence Flaherty	Human Investigation Committee	101 E. Alexandrine<br>Detroit, MI. 48201	USA	Protocol Version A<br>Amendment B<br>Amendment C	22-APR-10<br>23-SEP-10<br>24-JAN-11
201182	Rene Gonzalez	WIRB	3535 Seventh Ave, SW<br>Olympia, WA. 98508	USA	Protocol Version A<br>Amendment B<br>Amendment C	27-APR-10<br>14-SEP-10<br>28-DEC-10
201248	John Hainsworth	WIRB	3535 Seventh Ave<br>PO Box 12029<br>Olympia, WA. 98502	USA	Protocol Version A<br>Amendment B<br>Amendment C	29-DEC-09<br>10-AUG-10<br>Approval pending as of 04-MAR-11
201158	Thomas Hutson	Copernicus Group IRB	One Triangle Drive, Suite 100<br>Research Triangle Park, NC.	USA	Protocol Version A<br>Amendment B<br>Amendment C	27-OCT-09<br>07-JUL-10<br>17-NOV-10

vemurafenib (RO5185426) Roche Clinical Study Report - Protocol NO25026 - Research Report 1039652 2633

BRIM3 IRB List

CRTN	INVESTIGATOR	IRB/IEC Name	IRB/IEC Address	Country	Approval Dates	Date
201159	David Lawson	WIRB	3535 Seventh Ave, SW<br>Olympia, WA. 98508	USA	Protocol Version A<br>Amendment B<br>Amendment C	27-APR-10<br>31-AUG-10<br>07-DEC-10
200995	Gerald Linette	Washington University School of Medicine IRB	660 South Euclid Ave<br>Campus Box 8089<br>St. Louis, MO. 63110	USA	Protocol Version A<br>Amendment B<br>Amendment C	09-MAR-10<br>28-SEP-10<br>Approved by IRB but approval letter contained an error and site is working with IRB to fix
200996	Theodore Logan	IUPUI IRB	618 Union Building<br>620 Union Drive<br>Indianapolis, IN. 46202	USA	Protocol Version A<br>Amendment B<br>Amendment C	15-JAN-10<br>31-AUG-10<br>11-JAN-11
200997	Kim Margolin	WIRB	3535 Seventh Ave, SW<br>Olympia, WA. 98508	USA	Protocol Version A<br>Amendment B<br>Amendment C	02-FEB-10<br>18-AUG-10<br>28-DEC-10
200967	Stergios Moschos	Copernicus Group IRB	One Triangle Drive, Suite 100<br>Research Triangle Park, NC.	USA	Protocol Version A<br>Amendment B<br>Amendment C	10-FEB-10<br>07-JUL-10<br>17-NOV-10
200998	Steven O'Day	Copernicus Group IRB	One Triangle Drive, Suite 100<br>Research Triangle Park, NC.<br>27709	USA	Protocol Version A<br>Amendment B<br>Amendment C	27-OCT-09<br>07-JUL-10<br>17-NOV-10
200969	Anna Pavlick	NYU Medical Center IRB	550 First Avenue<br># VET 10th Floor, West Wing<br>New York, NY. 10016	USA	Protocol Version A<br>Amendment B<br>Amendment C	22-FEB-10<br>28-SEP-10<br>22-FEB-11

BRIM3 IRB List

CRTN	INVESTIGATOR	IRB/IEC Name	IRB/IEC Address	Country	Approval Dates	Date
200970	Antoni Ribas	Office for Protection of Research Studies	11000 Kinross Ave, Suite 102<br>Los Angeles, CA. 90095	USA	Protocol Version A<br>Amendment B<br>Amendment C	21-JAN-10<br>12-OCT-10<br>14-JAN-11
200971	Lynn Schuchter	U of Pennsylvania IRB	3624 Market St, Ste 301S<br>Philadelphia, PA. 19104-6006	USA	Protocol Version A<br>Amendment B<br>Amendment C	07-MAY-10<br>13-SEP-10<br>26-JAN-11
200991	Jeffrey Sosman	Vanderbilt University IRB	1313 21st Avenue South<br>504 Oxford House<br>Nashville, TN. 37232	USA	Protocol Version A<br>Amendment B<br>Amendment C	11-FEB-10<br>07-OCT-10<br>Approval pending as of 04-MAR-11
200999	Lynn Spitler	St. Mary's Medical Center Institutional Review Board	450 Stanyan St<br>San Francisco, CA. 94117	USA	Protocol Version A<br>Amendment B<br>Amendment C	27-JAN-10<br>31-AUG-10<br>19-JAN-11

vemurafenib (RO5185426) Roche Clinical Study Report - Protocol NO25026 - Research Report 1039652 2635

BRIM 7 IRB List

ETHICS AND IRB DETAILS

IRB/IEC Name	central / local	Chairman	Address	Country	CRTN(s)	Action	Date
Mount Zion Panel	Local	Andrew Parsa	Committee of Human Research Office of Research 3333 California Street, Suite 315 University of California San Francisco, CA 94118	USA	206577/Daud	Amendment F Approval	9/12/2012
						Amendment E Approval	2/14/2012
						Amendment D Approval	9/13/2011
						Amendment C Approval	7/12/2011
						Amendment B Approval	3/21/2011
Wayne Statue University Institutional Review Board	Local	James E. Puklin	Wayne State University 87 East Canfield, Second Floor Detroit, MI 48201	USA	245655/Flaherty	Amendment F Approval	8/24/2012
						Amendment E Approval	5/4/2012
University of Chicago Institutional Review Board	Local	Christopher Daugherty	University of Chicago McGiffert Hall, 2nd Floor 5751 S. Woodlawn Ave. Chicago, IL 60637	USA	206579/Gajewski	Amendment F Approval	10/10/2012
						Amendment E Approval	2/21/2012
						Amendment D Approval	9/14/2011
						Amendment C Approval	5/25/2011
						Amendment B Approval	2/25/2011

(RO5514041) Roche Clinical Study Report - Protocol NO25395 - Research Report 1057300 6476

BRIM 7 IRB List

Western Institutional Review Board	Central	Theodore D. Schultz	Western Institutional Review Board 3535 Seventh Avenue SW Olympia, WA 98502-5010	USA	206321/Gonzalez	Amendment F Approval	8/31/2012
						Amendment E Approval	1/13/2012
						Amendment D Approval	8/19/2011
						Amendment C Approval	4/25/2011
						Amendment B Approval	1/3/2011
Western Institutional Review Board	Central	Theodore D. Schultz	Western Institutional Review Board 3535 Seventh Avenue SW Olympia, WA 98502-5010	USA	240937/Hamid	Amendment F Approval	9/28/2012
						Amendment E Approval	1/20/2012
						Amendment D Approval	12/16/2011
						Amendment C Approval	12/16/2011
Indiana University Institutional Review Board	Local	N. Manchanda	Indiana University Office of Research Administration 980 Indiana Ave, Room 3315 Indianapolis, IN 46202-29215	USA	241099/Logan	Amendment F Approval	9/27/2012
						Amendment E Approval	4/25/2012
Peter MacCallum Cancer Centre Human Research Ethics Committee	Local	Dianne Snowden	Peter MacCallum Cancer Centre St Andrews Place East Melbourne, VIC 3002 Australia	AUS	206325/McArthur	Amendment F Approval	9/21/2012
		Namita Mazzitelli				Amendment E Approval	3/7/2012
		Sharon Reid				Amendment D Approval	9/16/2011
		Dianne Snowden				Amendment C Approval	5/6/2011

BRIM 7 IRB List

NYU School of Medicine Institutional Review Board	Local	Elan Czeisler	NYU School of Medicine 550 First Avenue New York, NY 10016	USA	206324/Pavlick	Amendment F Approval	9/25/2012
						Amendment E Approval	2/15/2012
						Amendment D Approval	8/30/2011
						Amendment C Approval	5/25/2011
						Amendment B Approval	2/3/2011
Vanderbilt University Institutional Review Board	Local	Steven L. Goudy	Vanderbilt University Institutional Review Board 1313 21st Avenue South 504 Oxford House Nashville, TN 37232-4315	USA	206319/Puzanov	Amendment F Approval	9/25/2012
						Amendment E Approval	2/22/2012
						Amendment D Approval	8/26/2011
						Amendment C Approval	7/13/2011
						Amendment B Approval	5/6/2011
Office of the Human Research Protection Program	Local	Fairooz Kabbinavar	Office of the Human Research Protection Program 11000 Kinross Avenue Los Angeles, CA 90095	USA	206318/Ribas	Amendment F Approval	9/28/2012
		Fairooz Kabbinavar				Amendment E Approval	2/14/2012
		Fairooz Kabbinavar				Amendment D Approval	8/5/2011
		Daniel Clemens				Amendment C Approval	5/19/2011
		Daniel Clemens				Amendment B Approval	2/4/2011

coBRIM IRB List

Listing and Details of Ethics Committees and Investigational Review Boards for Study GO28141

CRT No.	EC/IRB	Address
252958	Royal Brisbane and Women's HREC	Royal Brisbane and Women's Hospital, Butterfield Street, Herston, QLD, 4029, Australia
255130	Greenslopes Reseach and Ethics Committee	Greenslopes Private Hospital, Newdegate Street, Greenslopes, QLD, 4120, Australia
255084	Royal Perth HREC	Level 5, Colonical House, Royal Perth Hospital, Wellington Street, Perth, 6000, Australia
254430	Royal Brisbane and Women's HREC	Royal Brisbane and Women's Hospital, Butterfield Street, Herston, QLD, 4029, Australia
252561	Royal Adelaide Hospital HREC	Level 3, Hanson Institute IMVS Building, North Terrace, Adelaide, SA, 5000, Australia
252821	Royal Brisbane and Women's HREC	Royal Brisbane and Women's Hospital, Butterfield Street, Herston, QLD, 4029, Australia
253468	Royal Brisbane and Women's HREC	Royal Brisbane and Women's Hospital, Butterfield Street, Herston, QLD, 4029, Australia
252750	Royal Brisbane and Women's HREC	Royal Brisbane and Women's Hospital, Butterfield Street, Herston, QLD, 4029, Australia
252819	Tasmania Health and Medical HREC	301 Sandy Bay Campus, Office of Reseach Services, University of Tasmania, Private Bag 01, Hobart, TAS 7001, Australia
252692	Alfred Hospital EC	The Alfred Hospital, Ground Floor, Lina Pavilion, Commercial Road, Melbourne, VIC, 3004, Australia
252736	Bellberry HREC	229 Greenhill Road, Dulwich, SA, 5065, Australia
252814	HREC of Northern Territory Department of Health and Menzies School of Health Research	Royal Darwin Hospital Campus, Building 58, John Mathews Building, Rocklands Drive, Casuarina, NT, 0810, Australia
252824	Bellberry HREC	229 Greenhill Road, Dulwich, SA, 5065, Australia
252735	Royal Brisbane and Women's HREC	Royal Brisbane and Women's Hospital, Butterfield Street, Herston, QLD, 4029, Australia
252752	Royal Brisbane and Women's HREC	Royal Brisbane and Women's Hospital, Butterfield Street, Herston, QLD, 4029, Australia
252818	Tasmania Health and Medical HREC	301 Sandy Bay Road, Office of Reseach Services, University of Tasmania, Private Bag 01, Hobart, TAS 7001, Australia
253466	Bellberry HREC	229 Greenhill Road, Dulwich, SA, 5065, Australia
254367	EC Oberösterreich	Wager-Jauregg Weg15, 4020 Linz
256316	EC Niederösterreich	Landhausplatz 1, Haus 15B
254366	CEC Vienna, Medical University Vienna	Borschkegasse 8b/E 06, 1090 Vienna
255129	EC Krankenhaus Elisabethinen	Fadingerstrasse 1, 4020 Linz
256317	EC Salzburg	Seabstein-Stief Gasse 2, 5010 Salzburg
255077	Commission d'Ethique Biomédicale Hospitali-Facultaire de l'UCL	Avenue Hippocrate 55.14, Tour Harvey–Niveau 0 1200 Bruxelles Belgium
254421	Medische Commissie H.Hartziekenhuis Roeselare Menen vzw	Wilgenstraat 28800 Roeselare Belgium
254432	Comité d'Ethique de l'Institut Jules Bordet	Secrétariat de Chimiothérapie Boulevard de Waterloo 125 (3ième étage) 1000 Bruxelles Belgium

coBRIM IRB List

CRT No.	EC/IRB	Address
254362	Ethische Toetsingcommissie (ETC)	Stadsomvaart 11 3500 Hasselt Belgium
255076	Comité d'Ethique Hospitalo-Facultaire Universitaire de Liège	Domaine Universitaire du Sart Tilman, Bâtiment B35, Avenue de l'Hôpital 1 4000 Liège Belgium
253610	Ethisch Comité	Universitair Ziekenhuis Antwerpen Wilrijkstraat 10 2650 Edegem Belgium
254365	Ethisch Comité AZ Groeninge	Algemeen Ziekenhuis Groeninge, campus Maria's Voorzienigheid Loofstraat 43 8500 Kortrijk Belgium
256864	UBC BC Cancer Agency REB	Fairmont Medical Building, 750 West Broadway, Suite 902 Vancouver, B.C. Canada V5Z 1H8
256315	Comité d'éthique de la recherche du CHUQ	Hôpital Saint-François d'Assise<br>10, rue de L'Espinay, Local A0-124<br>Québec (Québec) G1L 3L5
256298	Ontario Cancer Research Ethics Board	MaRS Centre, South Tower, Suite 800<br>101 College Street Toronto, Ontario, Canada M5G 0A3
256831	Ontario Cancer Research Ethics Board	MaRS Centre, South Tower, Suite 800<br>101 College Street Toronto, Ontario, Canada M5G 0A3
254358	Hamilton Integrated Research Ethics Board	293 Wellington Street North, Suite 102<br>Hamilton ON L8L 8E7
254355	McGill IRB	3655 Promenade Sir William Osler, # 633<br>Montreal, Quebec<br>Canada H3G1Y6
256302	Ontario Cancer Research Ethics Board	MaRS Centre, South Tower, Suite 800<br>101 College Street Toronto, Ontario, Canada M5G 0A3
261705	UBC BC Cancer Agency REB	Fairmont Medical Building, 750 West Broadway, Suite 902<br>Vancouver, B.C. Canada V5Z 1H8
254357	Ottawa Hospital Research Ethics Board	MaRS Centre, South Tower, Suite 800<br>101 College Street Toronto, Ontario, Canada M5G 0A3
254368	Eticka komise Fakultni nemocnice Kralovske Vinohrady	Srobarova 50<br>100 34, Praha 10, Czech Rep.
254372	Eticka komise Nemocnice Na Bulovce	Budinova 2<br>180 01 Praha 8, Czech Rep.
252747	Eticka komise Vseobecne fakultni nemocnice v Praze	Na Bojisti 1<br>128 08 Praha 2, Czech Rep.
254375	Eticka komise Fakultni nemocnice Hradec Kralove	Sokolska 581<br>500 05 Hradec Kralove, Czech Rep.
252748	Eticka nemocnice Fakultni nemocnice Olomouc a LF UP	I.P. Pavlova 6<br>775 20 Olomouc, Czech Rep.
252749	Eticka komise Masarykova onkologickeho ustavu	Zluty kopec 7<br>656 53 Brno, Czech Rep.
254426	Eticka komise Fakultni nemocnice v Motole	V Uvalu 84<br>150 06 Praha 5, Czech Rep.
255124	Eticka komise Pardubicka krajska nemocnice, a.s.	Kyjevska 44<br>532 03, Pardubice, Czech Rep.

coBRIM IRB List

cobimetinib (RO5514041) Roche Clinical Study Report - Protocol GO28141 - Research Report 1060643 7486

CRT No.	EC/IRB	Address
254904	Eticka komise Fakultní nemocnice Ostrava	17. listopadu<br>708 52 Ostrava, Czech Rep.
257887	CPP Ouest 3	CHU La Milétrie, Pavilion le Blaye-Porte 9,<br>2 rue de la Milterie- BP 577<br>86021 Poitier Cedex
257517	CPP Ouest 3	CHU La Milétrie, Pavilion le Blaye-Porte 9,<br>2 rue de la Milterie- BP 577<br>86021 Poitier Cedex
254361	CPP Ouest 3	CHU La Milétrie, Pavilion le Blaye-Porte 9,<br>2 rue de la Milterie- BP 577<br>86021 Poitier Cedex
265143	CPP Ouest 3	CHU La Milétrie, Pavilion le Blaye-Porte 9,<br>2 rue de la Milterie- BP 577<br>86021 Poitier Cedex
255075	CPP Ouest 3	CHU La Milétrie, Pavilion le Blaye-Porte 9,<br>2 rue de la Milterie- BP 577<br>86021 Poitier Cedex
252623	CPP Ouest 3	CHU La Milétrie, Pavilion le Blaye-Porte 9,<br>2 rue de la Milterie- BP 577<br>86021 Poitier Cedex
253581	CPP Ouest 3	CHU La Milétrie, Pavilion le Blaye-Porte 9,<br>2 rue de la Milterie- BP 577<br>86021 Poitier Cedex
257505	CPP Ouest 3	CHU La Milétrie, Pavilion le Blaye-Porte 9,<br>2 rue de la Milterie- BP 577<br>86021 Poitier Cedex
252555	CPP Ouest 3	CHU La Milétrie, Pavilion le Blaye-Porte 9,<br>2 rue de la Milterie- BP 577<br>86021 Poitier Cedex
257502	CPP Ouest 3	CHU La Milétrie, Pavilion le Blaye-Porte 9,<br>2 rue de la Milterie- BP 577<br>86021 Poitier Cedex
256322	CPP Ouest 3	CHU La Milétrie, Pavilion le Blaye-Porte 9,<br>2 rue de la Milterie- BP 577<br>86021 Poitier Cedex
254902	CPP Ouest 3	CHU La Milétrie, Pavilion le Blaye-Porte 9,<br>2 rue de la Milterie- BP 577<br>86021 Poitier Cedex
258211	CPP Ouest 3	CHU La Milétrie, Pavilion le Blaye-Porte 9,<br>2 rue de la Milterie- BP 577<br>86021 Poitier Cedex

coBRIM IRB List

CRT No.	EC/IRB	Address
255863	CPP Ouest 3	CHU La Milétrie, Pavillon le Blaye-Porte 9, 2 rue de la Milterie- BP 577 86021 Poitier Cedex
252559	CPP Ouest 3	CHU La Milétrie, Pavillon le Blaye-Porte 9, 2 rue de la Milterie- BP 577 86021 Poitier Cedex
256325	Ethik-Kommission an der Medizinischen Fakultät der Eberhard-Karls-Universität und am Universitätsklinikum Tübingen	Gartenstr. 47 72074 Tübingen
254412	Ethik-Kommission an der Medizinischen Fakultät der Eberhard-Karls-Universität und am Universitätsklinikum Tübingen	Gartenstr. 47 72074 Tübingen
255083	Ethik-Kommission an der Medizinischen Fakultät der Eberhard-Karls-Universität und am Universitätsklinikum Tübingen	Gartenstr. 47 72074 Tübingen
252961	Ethik-Kommission an der Medizinischen Fakultät der Eberhard-Karls-Universität und am Universitätsklinikum Tübingen	Gartenstr. 47 72074 Tübingen
255081	Ethik-Kommission an der Medizinischen Fakultät der Eberhard-Karls-Universität und am Universitätsklinikum Tübingen	Gartenstr. 47 72074 Tübingen
254392	Ethik-Kommission an der Medizinischen Fakultät der Eberhard-Karls-Universität und am Universitätsklinikum Tübingen	Gartenstr. 47 72074 Tübingen
256836	Ethik-Kommission an der Medizinischen Fakultät der Eberhard-Karls-Universität und am Universitätsklinikum Tübingen	Gartenstr. 47 72074 Tübingen
255128	Ethik-Kommission an der Medizinischen Fakultät der Eberhard-Karls-Universität und am Universitätsklinikum Tübingen	Gartenstr. 47 72074 Tübingen
256314	Ethik-Kommission an der Medizinischen Fakultät der Eberhard-Karls-Universität und am Universitätsklinikum Tübingen	Gartenstr. 47 72074 Tübingen
254395	Ethik-Kommission an der Medizinischen Fakultät der Eberhard-Karls-Universität und am Universitätsklinikum Tübingen	Gartenstr. 47 72074 Tübingen
253512	Ethik-Kommission an der Medizinischen Fakultät der Eberhard-Karls-Universität und am Universitätsklinikum Tübingen	Gartenstr. 47 72074 Tübingen
254387	Ethik-Kommission an der Medizinischen Fakultät der Eberhard-Karls-Universität und am Universitätsklinikum Tübingen	Gartenstr. 47 72074 Tübingen
255053	Ethik-Kommission an der Medizinischen Fakultät der Eberhard-Karls-Universität und am Universitätsklinikum Tübingen	Gartenstr. 47 72074 Tübingen

coBRIM IRB List

CRT No.	EC/IRB	Address
256326	Ethik-Kommission an der Medizinischen Fakultät der Eberhard-Karls-Universität und am Universitätsklinikum Tübingen	Gartenstr. 47<br>72074 Tübingen
252967	Ethik-Kommission an der Medizinischen Fakultät der Eberhard-Karls-Universität und am Universitätsklinikum Tübingen	Gartenstr. 47<br>72074 Tübingen
255082	Ethik-Kommission an der Medizinischen Fakultät der Eberhard-Karls-Universität und am Universitätsklinikum Tübingen	Gartenstr. 47<br>72074 Tübingen
255011	Ethik-Kommission an der Medizinischen Fakultät der Eberhard-Karls-Universität und am Universitätsklinikum Tübingen	Gartenstr. 47<br>72074 Tübingen
252971	Ethik-Kommission an der Medizinischen Fakultät der Eberhard-Karls-Universität und am Universitätsklinikum Tübingen	Gartenstr. 47<br>72074 Tübingen
254413	Ethik-Kommission an der Medizinischen Fakultät der Eberhard-Karls-Universität und am Universitätsklinikum Tübingen	Gartenstr. 47<br>72074 Tübingen
257886	Ethik-Kommission an der Medizinischen Fakultät der Eberhard-Karls-Universität und am Universitätsklinikum Tübingen	Gartenstr. 47<br>72074 Tübingen
252969	Ethik-Kommission an der Medizinischen Fakultät der Eberhard-Karls-Universität und am Universitätsklinikum Tübingen	Gartenstr. 47<br>72074 Tübingen
256896	Ethics Committee for Clinical Pharmacology of the Medical Research Council	H-1051 Budapest, Zrinyi u. 3.
256897	Ethics Committee for Clinical Pharmacology of the Medical Research Council	H-1051 Budapest, Zrinyi u. 3.
256859	Ethics Committee for Clinical Pharmacology of the Medical Research Council	H-1051 Budapest, Zrinyi u. 3.
257397	Ethics Committee for Clinical Pharmacology of the Medical Research Council	H-1051 Budapest, Zrinyi u. 3.
252811	Rambam EC	Rambam Health Care Campus 6 Ha'Aliya Street POB 9602 Haifa 31096 Israel
254431	Rabin EC	Rabin Medical Center Beilinson Hospital: 39 Jabotinski St.,Petah Tikva
252828	Hadassah EC	Hadassah Medical Center Kiryat Hadassah, POB 12000 Jerusalem, 91120, Israel
252826	Sourasky EC	Tel Aviv Sourasky Medical Center address: 6 Weizmann Street Tel Aviv 64239, Israel
252757	Sheba EC	the Chaim Sheba Medical Center - Tel Hashomer 52621, Israel
252951	Soroka EC	Soroka University Medical Center POB 151, Beer-Sheva

coBRIM IRB List

CRT No.	EC/IRB	Address
252763	Comitato Etico Provinciale della Provincia di Brescia	Piazzale Spedali Civili n.1 25123 Brescia Italy
253588	Comitato Etico Dell'irccs Istituto Nazionale Per Lo Studio E La Cura Dei Tumori Fondazione Giovanni Pascale Di Napoli	Via MARIANO SEMMOLA 80131 Napoli- Italy
253607	Comitato Etico CESC dell'IRCCS Istituto Oncologico Veneto (IOV)	Via Gattamelata, 64 35128 Padova- Italy
254903	Comitato Etico Centrale IRCCS Lombardia	Giacomo Venezian, 1 20133 Milano - Italy
257793	COMITATO ETICO CENTRALE IRCCS con sede IFO	Via Elio Chianesi, 53 00144 Roma - Italy
253784	Comitato Etico dell' IRCCS Giovanni Paolo II di Bari	Via Orazio Flacco 65 70126 Bari- Italy
255078	Comitato Etico Area Vasta Sud Est	c/o U.O.C. Farmacia AOUS Viale Bracci 53100 Siena - Italy
255085	Comitato etico della provincia di Bergamo	Piazza Organizzazione Mondiale della Sanità (OMS), 1 24127 Bergamo - Italy
255086	Comitato Etico Provinciale Di Modena	Via Del Pozzo, 71<br>41100 Modena - Italy
253609	Comitato Etico Regionale Della Liguria	Largo Rosanna Benzi 10<br>16132 Genova- Italy
254363	Comitato Etico Di Area Vasta Romagna Di Cesena E Istituto Scientifico Romagnolo Per Lo Studio E La Cura Dei Tumori Di Meldola (FC)	Via Piero Maroncelli, 40<br>47014 Meldola (FC) - Italy
255854	Comitato etico degli IRCCS Istituto Europeo di Oncologia e Centro Cardiologico Monzino	Via Ripamonti 435<br>20141 Milano - Italy
252548	METC AzM/UM	Maastricht UMC, dep. METC AzM/UM, P. debeyelaan 25, 6229 HX Maastricht, the Netherlands
253782	METC AzM/UM	Maastricht UMC, dep. METC AzM/UM, P. debeyelaan 25, 6229 HX Maastricht, the Netherlands
256404	METC AzM/UM	Maastricht UMC, dep. METC AzM/UM, P. debeyelaan 25, 6229 HX Maastricht, the Netherlands
253783	Health and Disability Ethics Committees	1 the Terrace, PO Box 5013, Wellington, 6011, New Zealand
253582	Health and Disability Ethics Committees	1 the Terrace, PO Box 5013, Wellington, 6011, New Zealand
254425	REK sør-øst	Nydalen allé 37B, 0484 Oslo
254414	REK sør-øst	Nydalen allé 37B, 0484 Oslo
257401	Ethics Committee at the"Russian Oncology Scientific Center n.a. N.N. Blokhin"	Khashirskoe shosse,24, Moscow, 115478
258059	Ethics Committee at the Budgetary Healthcare Institution of the Omsk Region "Clinical Oncology Dispensary"	Zavertyaeva ul. 9, korp. 1, Omsk, 644013
257399	Ethics Committee at the State Territorial Budgetary Healthcare Institution "Altai Territory Oncology Dispensary"	Nikitina ul. 77, Barnaul, 656049

coBRIM IRB List

CRT No.	EC/IRB	Address
257400	Ethics Committee at the Moscow State Budgetary Healthcare Institution "Moscow City Oncology Hospital # 62"	Istra settlement, 27, Krasnogorsk district, Moscow region, 143423
257398	Ethics Committee at the Stavropol Territory State Budgetary Healthcare Institution "Pyatigorsk Oncology Dispensary"	Kalinina pr. 31, Pyatigorsk, 357500
254415	CEIC Área 4 - Hospital Universitario Ramón y Cajal	Ctra. De Colmenar Viejo, km. 9,1 28034, Madrid
254417	CEIC Área 4 - Hospital Universitario Ramón y Cajal	Ctra. De Colmenar Viejo, km. 9,1 28034, Madrid
254427	CEIC Área 4 - Hospital Universitario Ramón y Cajal	Ctra. De Colmenar Viejo, km. 9,1 28034, Madrid
254423	CEIC Área 4 - Hospital Universitario Ramón y Cajal	Ctra. De Colmenar Viejo, km. 9,1 28034, Madrid
254418	CEIC Área 4 - Hospital Universitario Ramón y Cajal	Ctra. De Colmenar Viejo, km. 9,1 28034, Madrid
252952	CEIC Área 4 - Hospital Universitario Ramón y Cajal	Ctra. De Colmenar Viejo, km. 9,1 28034, Madrid
254428	CEIC Área 4 - Hospital Universitario Ramón y Cajal	Ctra. De Colmenar Viejo, km. 9,1 28034, Madrid
252816	CEIC Área 4 - Hospital Universitario Ramón y Cajal	Ctra. De Colmenar Viejo, km. 9,1 28034, Madrid
255383	Regionala etikprövningsnämnden i Lund	Box 133, 221 00 Lund, Sweden
255385	Regionala etikprövningsnämnden i Lund	Box 133, 221 00 Lund, Sweden
255384	Regionala etikprövningsnämnden i Lund	Box 133, 221 00 Lund, Sweden
254905	Regionala etikprövningsnämnden i Lund	Box 133, 221 00 Lund, Sweden
254376	Kantonale Ethikkomission Bern	Postfach 56 3010 Bern
252950	Kantonale Ethikkommission Zürich	Stampfenbachstrasse 121 (Eingang Georgengasse 6 benützen!) 8090 Zürich
252753	NRES Comittee London - Brent	80 London Road, Skipton House, London, SE1 6LH
255856	NRES Comittee London - Brent	80 London Road, Skipton House, London, SE1 6LH
255126	NRES Comittee London - Brent	80 London Road, Skipton House, London, SE1 6LH
254378	NRES Comittee London - Brent	80 London Road, Skipton House, London, SE1 6LH
252825	NRES Comittee London - Brent	80 London Road, Skipton House, London, SE1 6LH

coBRIM IRB List

CRT No.	EC/IRB	Address
255052	NRES Comittee London - Brent	80 London Road, Skipton House, London, SE1 6LH
252754	NRES Comittee London - Brent	80 London Road, Skipton House, London, SE1 6LH
252755	NRES Comittee London - Brent	80 London Road, Skipton House, London, SE1 6LH
256313	NRES Comittee London - Brent	80 London Road, Skipton House, London, SE1 6LH
256312	NRES Comittee London - Brent	80 London Road, Skipton House, London, SE1 6LH
254381	NRES Comittee London - Brent	80 London Road, Skipton House, London, SE1 6LH
255113	NRES Comittee London - Brent	80 London Road, Skipton House, London, SE1 6LH
255127	NRES Comittee London - Brent	80 London Road, Skipton House, London, SE1 6LH
256835	NRES Comittee London - Brent	80 London Road, Skipton House, London, SE1 6LH
255080	NRES Comittee London - Brent	80 London Road, Skipton House, London, SE1 6LH
254429	St. Lukes Hospital and Health Network IRB	801 Ostrum Street<br>Bethlehem, PA 18015
254364	California Pacific Medical Center IRB	2200 Webster Street, 5th Floor<br>San Francisco, CA 94115
252546	Copernicus Group IRB	1 Triangle Drive, Suite 100<br>Research Triangle Park, NC 27709
252957	University of Louisville IRB	Research Triangle Park, NC
256893	UC Davis IRB	2921 Stockton Blvd<br>Suite 1400, Room 1429<br>Sacramento, CA 95817
255848	WIRB	Western Institutional Review Board 3535 7th Avenue SW Olympia, WA 98502
253109	Rhode Island Hospital IRB	RI IRB #2<br>593 Eddy Street<br>Providence, RI 02903
252690	University of California San Diego Human Research Protections Program	9500 Gilman Drive, Mail Code 0052<br>La Jolla CA 92093
254890	University of Kansas IRB	3901 Rainbow Boulevard<br>Kansas City, KS 66160
253110	Dartmouth Commitee for the Protection of Human Subjects (CPHS) IRB	63 South Main Street, Room 302<br>Hanover, NH 03755
256833	WIRB	Western Institutional Review Board 3535 7th Avenue SW Olympia, WA 98502
252949	Thomas Jefferson University	1015 Chestnut Street, Suite 1100<br>Philadelphia, PA 19107
254899	University of Chicago IRB	McGiffert Hall, 2nd Floor<br>5751 South Woodlawn Avenue<br>Chicago, IL 60637

coBRIM IRB List

CRT No.	EC/IRB	Address
252481	Copernicus Group IRB	1 Triangle Drive, Suite 100<br>Research Triangle Park, NC 27709
259614	WIRB	Western Institutional Review Board 3535 7th Avenue SW Olympia, WA 98502
255079	WIRB	Western Institutional Review Board 3535 7th Avenue SW Olympia, WA 98502
252621	Kaiser Permanente IRB - Northern California	1800 Harrison Street, 16th Floor<br>Oakland CA, 94612
255123	Chesapeake IRB	7063 Columbia Gateway Drive, Suite 110<br>Columbia, MD 21046
255115	Northwestern University IRB	750 N. Lake Shore Drive - 7th Floor<br>Chicago IL, 60611
256321	WIRB	Western Institutional Review Board 3535 7th Avenue SW Olympia, WA 98502
252547	Washington University in St. Louis IRB	660 S. Euclid Ave., Box 8089<br>St. Louis MO 63110
257373	Institutional Review Board of Mount Sinai Medical Center	4300 Alton Roead<br>Miami Beach, FL 33140
256320	TriHealth Institutional Review Board	10498 Montgomery Road, Suite A Cincinnati OH 45242
255119	Mayo Clinic IRB - Rochester	200 First Street SW<br>201 Building Rm 4-60<br>Rochester, MN 55905
252689	Copernicus Group IRB	1 Triangle Drive, Suite 100<br>Research Triangle Park, NC 27709
254356	Copernicus Group IRB	1 Triangle Drive, Suite 100<br>Research Triangle Park, NC 27709
256894	WIRB	Western Institutional Review Board 3535 7th Avenue SW Olympia WA 98502
254891	Biomedical Research Alliance of New York, LLC	One Park Avenue, 6th Fl New York, NY 10016
252691	MD Anderson Cancer Center Orlando IRB	1414 Kuhl Avenue Orlando, FL 32806
254359	Vanderbilt University Medical Center IRB	1313 21st Avenue South, Ste 504 OH Nashville, TN 37232
254900	UCLA Medical Center IRB	11000 Kinross Ave, Ste. 211 Box 951694 Los Angeles, CA 90095
252734	University of Minnesota Institutional Review Board	420 Delaware Street SE D-528 Mayo Memorial Building Minneapolis, MN 55455
252618	Duke University Health System Institutional Review Board	2424 Erwin Road, Suite 405<br>Durham, NC 27710
253467	Hartford Hospital IRB	80 Seymour Street P.B. Box 5037<br>Hartford, CT 06102
253107	Stanford University Administrative Panel on Human Subjects in Medical Research	1501 South California Avenue<br>Palo Alto, CA 94304
256305	WIRB	Western Institutional Review Board 3535 7th Avenue SW Olympia, WA 98502
253108	Copernicus Group IRB	1 Triangle Drive, Suite 100 Research Triangle Park, NC 27709
